# Effect of autologous dendritic cell cytokine-induced killer on refractory metastatic colorectal cancer: a matched case–control comparative study

**DOI:** 10.3389/fimmu.2024.1329615

**Published:** 2024-02-27

**Authors:** Sheng-Chi Chang, Tao-Wei Ke, William Tzu-Liang Chen, Weoi-Cherng Shyu, Long-Bin Jeng

**Affiliations:** ^1^ Division of Colorectal Surgery, Department of Surgery, China Medical University Hospital, Taichung, Taiwan; ^2^ Cell Therapy Center, China Medical University Hospital, Taichung, Taiwan; ^3^ Division of Colorectal Surgery, Department of Surgery, China Medical University Hsinchu Hospital, Hsinchu, Taiwan; ^4^ Translational Medicine Research Center, Drug Development Center and Department of Neurology, China Medical University and Hospital, Taichung, Taiwan; ^5^ Organ Transfer Center, China Medical University Hospital, Taichung, Taiwan

**Keywords:** dendritic cell-based adoptive cell transfer, DC-CIK, refractory metastatic colorectal cancer, survival, comparison

## Abstract

**Background:**

Patients with metastatic colorectal cancer (mCRC) who are refractory to two or more lines of systemic chemotherapy have limited therapeutic options. The aim of this study was to evaluate the effect of autologous dendritic cell cytokine-induced killer (DC-CIK) transfer on the survival of patients with mCRC who are refractory or intolerant to at least two lines of systemic chemotherapies.

**Methods:**

A matched case–control comparative study was conducted with patients who received DC-CIK immunotherapy in addition to standard chemotherapy (cases) and those with standard chemotherapy alone (controls). The primary objective was to compare the duration of oncologic survival, including overall survival (OS) and progression-free survival (PFS), between the two groups.

**Results:**

A total of 27 cases and 27 controls were included. The median OS in the DC-CIK case group was 18.73 ± 5.48 months, which was significantly longer than that in the control group (14.23 ± 1.90 months, *p* = 0.045). However, there was no significant difference in PFS between the two groups (*p* = 0.086). Subgroup analysis showed that in patients with liver or extra-regional lymph node metastasis, DC-CIK cases had longer OS than controls (17.0 vs. 11.87 months, *p* = 0.019; not match vs. 6.93 months, *p* = 0.002, respectively). In patients with Eastern Cooperative Oncology Group (ECOG) scale 0 or wild RAS/BRAF, DC-CIK cases showed a significant increase in OS duration compared to controls (28.03 vs. 14.53 months, *p* = 0.038; 18.73 vs. 11.87 months, *p* = 0.013, respectively).

**Conclusions:**

The addition of autologous DC-CIK to standard chemotherapy had a positive effect on OS of patients with refractory mCRC, especially those with liver or extra-regional lymph node metastasis, ECOG = 0, and wild RAS/BRAF status.

## 1 Introduction

Once patients with metastatic colorectal cancer (mCRC) were refractory to the first two lines of systemic chemotherapy, the subsequent therapeutic options became fewer. Regorafenib and TAS-102 are approved by the Food and Drug Administration in this setting, but survival benefit after these drugs remains poor, with the median overall survival (OS) being only 7.8 to 8.8 months (1, 2). In addition, the adverse effects of these late-line systemic chemotherapies are an important problem that can result in discontinuation. Furthermore, standard therapies may not even be applicable to these patients with chemo-refractory mCRC due to their poor general health (3).

Therefore, new strategies should be considered to improve the clinical practices during the chemo-refractory period. Because these patients are in an immunosuppressed state after sequential standard antitumor therapies, such as chemotherapy, surgery, or radiotherapy, a combined immunotherapy strategy for such insufficient immunity should be considered. As a promising immunotherapy for cancer, adoptive cell transfer (ACT) is achieved by *in vitro* expansion of autologous tumor-specific effector cells, such as cytokine-induced killer (CIK), dendritic cell vaccine (DCV), or dendritic cell-cytokine-induced killer (DC-CIK), which are then transferred back to patients. Though ACT is a more complex approach for cancer treatment, it offers more personalized approaches and has been proven as an effective immunotherapy for refractory lymphoblastic leukemia (4) and metastatic melanoma (5).

In the past decade, ACT by DCV or DC-CIK has been widely used in CRC treatment, and its clinical advantage has been reported by altering the patient’s immune responses and eradicating the circulating residual tumor cells (6, 7). Dendritic cells (DCs) are the strongest antigen-presenting cells to regulate and generate the memory T-lymphocyte response against CRC micro-lesions (7–9). Two meta-analysis studies demonstrated that DC-CIK immunotherapy combined with chemotherapy was effective in patients with CRC with longer survival and alleviated the adverse effects caused by chemotherapy (10, 11). However, to our knowledge, the effect of ACT in chemo-refractory patients with mCRC is rarely mentioned. Therefore, we designed a matched case–control comparative study, comparing autologous DC-CIK combined with standard chemotherapy (case arm) versus standard chemotherapy alone (control arm) in patients with mCRC who are refractory or intolerant to at least two lines of systemic chemotherapy.

## 2 Materials and methods

### 2.1 Study design and study population

Based on our institution’s prospective patient registry database, a matched case–control comparative design was implemented to address the objective of this study. Patients who underwent late-line chemotherapy (≥3 lines) for refractory mCRC between 2015 and 2022 at the China Medical University Hospital were included. Patients with a history of other malignancies were excluded. All eligible patients’ disease status was measured according to carcinoembryonic antigen (CEA) level and RECIST1.1, and their survival duration was recorded. This study was approved by the Institutional Review Board of the China Medical University Hospital, Taichung, Taiwan (CMUH111-REC3-191).

### 2.2 Case selection, inclusion, and exclusion criteria

Cases were defined as the patients with additional DC-CIK immunotherapy in the standard systemic chemotherapy for refractory mCRC in our recorded period. We excluded individuals who did not receive at least four doses of DC-CIK or who discontinued ACT therapy in 4 weeks.

### 2.3 Control definition, inclusion, and exclusion criteria

For each case, the group of controls consisted of all eligible individuals with standard Regorafenib treatment for refractory mCRC in the same period. Individuals who used Regorafenib for less than 4 weeks or an initial dose of less than 80 mg were excluded.

### 2.4 Matching variables

Controls were individually matched 1:1 with each case for the following five covariates: age ( ± 15 years), previous chemotherapy lines, Eastern Cooperative Oncology Group (ECOG) performance status (PS) score, RAS and BRAF status, and primary tumor location.

### 2.5 Assessment of observed outcomes

Our primary objective was to compare cases and controls in terms of duration of oncologic survival, including OS (time from the first dose of DC-CIK or the first dose of Regorafenib to the date of documented patient death) and progression-free survival (PFS; time from the first dose of DC-CIK or Regorafenib therapy to the date of documented progress in tumor size enlargement or new metastatic lesions according to RECIST criteria).

### 2.6 Preparation and administration of DC-CIK

#### 2.6.1 Preparation of tumor antigen

Fresh resected tumor specimens of at least 1 cm^3^ are obtained by sterile methods in an operating room and stored temporarily at 4°C and then the non-tumor part is removed manually. After six freeze–thaw cycles, the autologous tumor antigen lysate is collected and then cryopreserved.

#### 2.6.2 DC preparation

Peripheral blood mononuclear cells (PBMCs) are harvested from peripheral blood through apheresis and separated by Ficoll-Paque gradient centrifugation. Then, the monocytes are cultured, adhered, purified, cryo-frozen, and stored. For DC cell deformation, adhered monocytes are stimulated *in vitro* by granulocyte-macrophage colony-stimulating factor (GM-CSF, 800 U/mL), interleukin 4 (IL-4, 500 U/mL), and tumor necrotic factor alpha (TNF-α, 10 ng/mL) for 7 days to generate autologous immature DCs. Then, the immature DCs were incubated with autologous tumor antigen lysates for 48 h to obtain mature DCs. Finally, the DC phenotype (CD3, CD86, and HLA-DR) is analyzed by flow cytometry.

#### 2.6.3 CIK preparation

PBMCs are activated *in vitro* with the recombinant cytokines interleukin 2 (IL-2, 1,000 U/mL), interferon gamma (IFN-γ, 1,000 U/mL), and CD3 monoclonal antibody at 50 ng/dL for 7 to 10 days to generate CIK. Next, the CIK phenotypes (CD3/CD8, CD3/CD56, and CD3/NKG2D) are characterized by flow cytometry.

#### 2.6.4 DC-CIK preparation

The autologous mature DCs are mixed with cultured CIKs at a proportion of 1:30–1:50 for 72 h. Among cultured DC-CIK cells, the proportion of CD3 cells should reach less than 2%. The proportion of CD86 and HLA-DR cells should reach greater than 50%. For the identity of DC-CIK, the proportion of CD3/CD8 should be more than 40%. For the potency of DC-CIK, the proportion of CD3/CD56 and CD3/NKG2D cells should reach greater than 4% and 40%, respectively. Finally, antigen-specific DC-CIKs contain at least 1 × 10^9^ cells in each tube.

#### 2.6.5 Administration

The tube of DC-CIKs are diluted to 250 mL of saline and administered intravenously over 2–3 h on days 1, 5, and 9 for the first cycle and then repeatedly for the second cycle after the standard treatment such as chemotherapy. The interval of every cycle was approximately 3 to 4 weeks. The total of six doses of DC-CIK will be completed within 3 months since the first administration.

### 2.7 Oncological follow-up program

Both groups had a regular follow-up program, involving physical examination, serum tumor markers (CEA andCA19-9), and chest/abdomen/pelvic computed tomography every 3 months. Colonoscopy was performed annually. Positron emission tomography (PET) was performed according to the physician’s clinical judgment. Once tumor progression was noted from the image, the current regimen of systemic treatment (Regorafenib or intravenous chemotherapy or ACT immunotherapy) was stopped and changed.

### 2.8 Statistical analysis

We reported categorical variables as percentages while continuous variables were reported as mean ( ± standard deviation). The chi-square test was used for comparisons of categorical data, while the Mann–Whitney test was used to compare the continuous variables. OS and PFS were estimated using the Kaplan–Meier method, and cumulative survival between groups was compared using the logarithmic rank test. All variables with *p* < 0.05 were considered statistically significant.

## 3 Results

### 3.1 Characteristics of case and control samples

According to the patient allocation algorithm (**Supplementary 1**), 27 DC-CIK cases and 27 control subjects were enrolled. The mean age was 59.9 ± 6.9 and 60.7 ± 9.1 years for cases and controls, respectively. In both cases and controls, the distributions of previous chemotherapy lines, ECOG score, proportion of RAS or BRAF mutation, and primary tumor location were the same. In DC-CIK cases, 20 (74%) patients received chemo-target combination therapy and 7 (26%) patients had Regorafenib or Lonsurf monotherapy. In the control group, combined chemo-target therapy was used in 14 (52%) patients and Regorafenib monotherapy was prescribed in 13 (48%) patients. The proportion of patients with multiple site metastases, primary tumor resection, and prior anti-VEGF or anti-EGFR therapy, and gender did not differ between cases and controls (**Table 1**). We also observed that DC-CIK cases had a significantly higher rate of liver metastasis, but other organ metastases, such as extra-regional lymph nodes (ERLNs), peritoneum, and lung, had no difference between groups.

**Table 1:** Demographic data of patients treated with combined DC-CIK and chemotherapy (cases) or standard Regorafenib therapy (controls) for refractory metastatic colorectal cancer.

	All patients			Matched patients		
	DC-CIK cases	Controls	*p*-value	DC-CIK cases	Controls	*p*-value
Cases	32	152		27	27	
*Age	57.6 ± 8.3	59.5 ± 11.9	0.386	59.9 ± 6.9	60.7 ± 9.1	0.700
Female	17 (53.1)	75 (49.3)	0.697	13 (48.1)	11 (40.7)	0.584
*ECOG 0/1/2	12 (37.5)/16 (50.0)/4 (12.5)	84 (55.3)/63 (41.4)/5 (3.3)	0.036	13 (48.1)/14 (51.9)/0	13 (48.1)/14 (51.9)/0	1.000
*Primary tumor Right/Left colon/Rectum	6 (18.8)/16 (50.0)/10 (31.3)	36 (23.7)/53 (34.9)/63 (41.4)	0.184	3 (11.1)/14 (51.9)/10 (37.0)	3 (11.1)/14 (51.9)/10 (37.0)	1.000
*RAS or BRAF mutation	19 (59.4)	93 (61.2)	0.849	14(51.9)	14(51.9)	1.000
*Previous C/T lines 2/3/4/≥5	25 (78.1)/5 (15.6)/2 (6.3)/0	48 (31.6)/61 (40.1)/22 (14.5)/21 (13.8)	0.002	21 (77.8)/5 (18.5)/1 (3.7)/0	21 (77.8)/5 (18.5)/1 (3.7)/0	1.000
Multiple metastasis	27 (84.4)	75 (49.3)	0.001	24 (88.9)	19 (70.4)	0.091
Primary resection	30 (93.8)	135 (88.8)	0.404	26 (96.3)	24 (88.9)	0.299
Prior anti-VEGF Tx	29 (90.6)	134 (88.2)	0.690	24 (88.9)	19 (70.4)	0.091
Prior EGFR antibody Tx	11 (34.4)	62 (40.8)	0.500	11 (40.7)	13 (48.1)	0.584

Values are presented as mean ± standard deviation or numbers (%). *means five covariates for case–control matching comparison.

DC-CIK, dendritic cell–cytokine-induced killer; ECOG, Eastern Cooperative Oncology Group; C/T, chemotherapy; Tx, therapy; VEGF, vascular endothelial growth factor; EGFR, epidermal growth factor receptor.

### 3.2 Cell phenotype of DCs and DC-CIK

The results of flow cytometry for the DC and DC-CIK phenotype from 27 DC-CIK cases are shown in **Supplementary 2**, and the average data (mean ± SD) were presented as follows: The means of the DC phenotype were 0.23 ± 0.27% for CD3, 83.5 ± 13.6% for CD86, and 95.0 ± 3.4% for HLA-DR. The means of the DC-CIK phenotype were 77.1 ± 12.1% for CD3/CD8, 9.8 ± 7.8% for CD3/CD56, and 86.1 ± 11.2% for CD3/NKG2D. All flow cytometry data for cell phenotypes met the criterion of DC-CIK preparation.

### 3.3 Toxicity of DC-CIK

In 32 cases receiving more than four doses of DC-CIK therapy, no serious acute allergic reactions such as anaphylaxis or shock or autoimmune sequelae were observed. The most common adverse effects were fever in two cases (6.3%) and general malaise in two cases (6.3%). The overall adverse effect rate was 15.6% and no grade 3–4 toxicity was documented.

### 3.4 Chemotherapy-induced neutropenia

A total of 21 patients (38.9%) experienced chemotherapy-induced neutropenia (CIN) after third-line chemotherapy in our study, 8 (29.6%) in DC-CIK cases and 13 (48.1%) in controls (*p* = 0.163). Grade 3 or 4 CIN was observed in eight patients (14.8%) in our study, and it occurred in fewer patients in the DC-CIK case group compared to controls (3, 11.1% vs. 5, 18.5%, *p* = 0.444).

### 3.5 Tumor response

According to RECISTs criteria, two patients revealed a partial response in DC-CIK cases. A total of 17 cases and 15 controls maintained stable disease after treatment for 3 months. The disease control rate was 70.4% in DC-CIK cases and 55.6% in controls, with no statistically significant difference (*p* = 0.232). We measured CEA change from baseline to 8 weeks after the first dose of DC-CIK or standard chemotherapy administration, and the results revealed that DC-CIK cases had six patients (22.2%) with decreased CEA intervals of more than 50% and five patients (18.5%) with decreased CEA intervals of less than 50%. However, the control group only presented two (7.4%) and four (14.8%) patients who experienced decreased CEA intervals of more than or less than 50%, respectively (**Supplementary 3**).

### 3.6 Survival results

**Figure 1:**
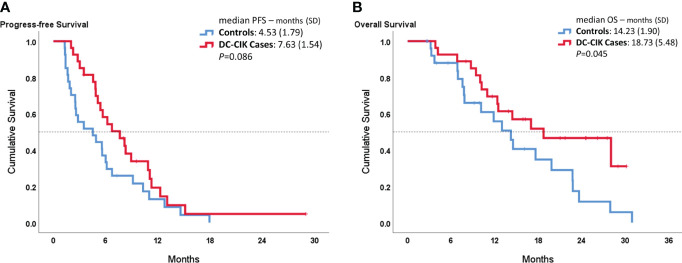
Kaplan–Meier survival curves comparing DC-CIK cases and controls for **(A)** progression-free survival (PFS) and **(B)** overall survival (OS).

In **Figure 1**, the median PFS showed 7.63 ± 1.54 months in DC-CIK cases, compared to 4.53 ± 1.54 months in controls with no statistically significant difference (*p* = 0.086). However, the DC-CIK cases had 18.73 ± 5.48 months of median OS, which was statistically significantly longer than the controls (14.23 ± 1.90 months, *p* = 0.045).

**Figure 2:**
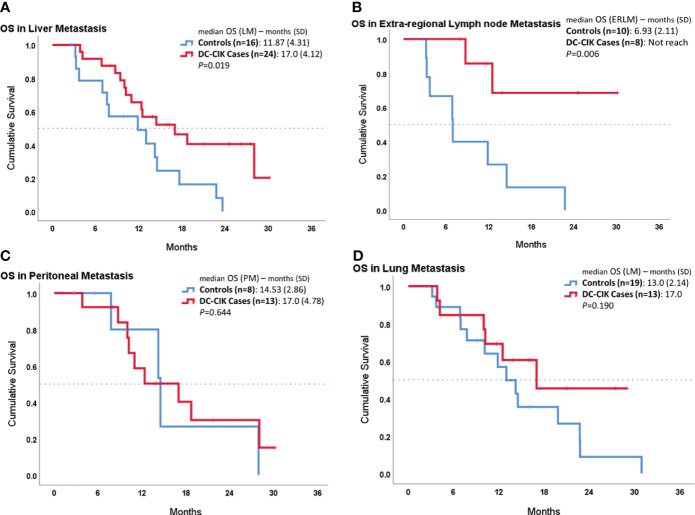
Subgroup survival analysis in patients with different organ metastases by comparing DC-CIK cases and controls. **(A)** Patients with liver metastasis. **(B)** Patients with extra-regional lymph node (ERLN) metastasis. **(C)** Patients with lung metastasis. **(D)** Patients with peritoneal metastasis.

In the subgroup analysis, we compared OS according to the different metastatic sites, as shown in **Figure 2**. In patients with liver metastasis, DC-CIK cases demonstrated statistically significantly increased OS compared to controls (17.0 ± 4.12 vs. 11.87 ± 4.31 months, *p* = 0.019). In patients with ERLN metastases, the duration of median OS was also statistically significantly longer in DC-CIK cases (did not reach vs. 6.93 ± 2.11 months, *p* = 0.002). In contrast, once distal metastasis involved the peritoneum or the lungs, the OS did not reveal any difference.

**Figure 3:**
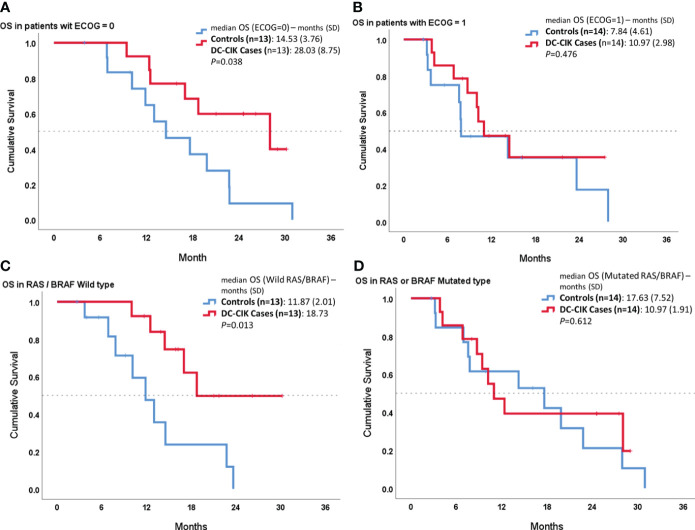
Subgroup survival analysis according to Eastern Cooperative Oncology Group (ECOG) performance status and RAS/BRAF status. Kaplan–Meier survival curves comparing DC-CIK cases and controls for **(A)** patients with ECOG score 0, **(B)** patients with ECOG score 1, **(C)** patients with both RAS/BRAF wild type, and **(D)** patients with RAS or BRAF mutation.

In the other subgroup analysis, we compared OS between cases and controls according to ECOG PS or RAS/BRAF status (**Figure 3**). In both patient subgroups with an ECOG score of 0 or wild RAS/BRAF, DC-CIK cases showed a statistically significantly superior OS, compared to controls (*p* = 0.038 and *p* = 0.013). In contrast, there was no difference seen in OS duration between DC-CIK cases and controls in the patient subgroup with an ECOG score of 1 or with a mutated RAS/BRAF status.

## 4 Discussion

Our study is the first retrospectively matched case–control comparative clinical study to evaluate the effect of DC-CIK combined with systemic chemotherapy in patients with highly pretreated mCRC. It revealed three important findings. First, the combination of DC-CIK in refractory mCRC treatment prolonged OS, compared to chemotherapy alone. Second, patients with liver or ERLN metastasis demonstrated statistically significantly longer survival in DC-CIK cases than controls. Finally, adding DC-CIK in the systemic treatment of patients with RAS/BRAF wild type or better PS (ECOG = 0) presented predominantly increased OS compared to those treated with chemotherapy alone.

In patients exploring multidisciplinary treatment for mCRC, immunosuppression was often apparent, such as the heavily pretreated patients in our study. The immunity dysfunction was related to tumor-induced modulation and bone marrow suppression from multiple chemotherapies. In the tumor microenvironment, DCs obtained from such heavily treated patients with cancer appear to be phenotypically and functionally defective and impaired by several mechanisms, such as the release of tumor-derived cytokines, tumor metabolites, and tumor immune escape pathway (12). The disturbed immunity environment results in cancer progression and shortens patient survival (13). Therefore, DC-based adoptive cell transfusion seems to be an alternative therapeutic approach to correct abnormal DC function and improved the immune reaction in the tumor microenvironment of the recovery patients. Studies demonstrated that active DC-CIK not only induces adaptive antitumor immunity (14) but also stimulates tumor antigen-specific cytotoxic T lymphocytes to recognize and eliminate malignant cells (15). Two prospective phase II trials showed that the DC-CIK can induce the proliferation of autologous tumor-specific T cells and the expression of IFN-γ, IL-2, and TNF-α in CD4+ T cells. Both trials concluded a positive association between elicited immune reaction and patient survival (16, 17).

CIN is a common and potentially fatal complication of myelosuppressive chemotherapy, which presented in approximately 46% of patients with refractory mCRC (18). It can have short- or long-term impacts on treatment plans, leading to unfavorable disease control and survival, and may result in patients missing potential opportunities for cure due to the severe consequences of CIN (19). However, studies have shown that DC-CIK immunotherapy combined with chemotherapy can enhance the immune response to tumors by providing a strong antigen-presenting ability and inherent cytotoxic ability (15–17). Therefore, the synergistic use of DC-CIK can overcome chemotherapy-induced myelosuppression and enhance the immune response. A previous double-blind randomized study showed that the combination of DC-CIK with advanced non-small cell lung cancer (NSCLC) therapy resulted in fewer cases with leukopenia and bone marrow suppression than the control group (20). Our study also presented fewer patients experiencing CIN in DC-CIK cases compared to controls (29.6% vs. 48.1%). However, further research is needed in this field to determine its specific mechanism of action and effects.

For CRC treatment, several studies have demonstrated the objective clinical benefit of DC-CIK in adjuvant therapy for patients with CRC who had completed tumor resection (21–24). Du et al. (21) conducted a comparative study in 253 patients who underwent primary resection of advanced CRC (TNM stages III and IV) and reported that DC-based ACT combined with chemotherapy significantly improved OS, compared to postoperative chemotherapy alone. However, in the same study (21), in patients with early CRC (stage II), postoperative use of DC-based ACT did not show any survival benefit. Xie et al. (22) also showed similar postoperative survival advantage and noted fewer tumor recurrence by using DC-CIK in patients with advanced CRC combined with first-line therapy. Subsequently, a phase II clinical trial was created in 2018 to evaluate the efficiency of DCV use after complete resection of CRC liver metastases and indicated a clear tendency to fewer and later tumor relapses in DCV patients (25.25 vs. 9.53 months) (24). These clinical benefits of DC-based ACT may be related to their strong antitumor activities through immuno-modulation and may be able to eradicate residual circulating tumor cells in advanced cancer following tumor resection.

For patients with multiple metastatic CRC who are not eligible for complete resection, the survival benefit of DC-based immunotherapy was controversial, although both peripheral tumor-specific T lymphocytes and immune-related cytokines increased significantly after autologous cell transfusion (9, 17, 25). Lin et al. conducted a prospective study to evaluate the effects of autologous DC-CIK treatment in 134 patients with unresectable CRC or relapsed/metastatic CRC. They found that a combination of DC-CIK and first-line chemotherapy could induce a greater T-cell response and significantly prolong survival (17). In this study, the median PFS and OS in the DC-CIK treatment group were 8.8 months (95% CI 8.4–9.1) and 14.7 months (95% CI 13.9–15.5), respectively, which showed statistically significant improvement compared to the PFS and OS in the control group. Our study of patients with refractory mCRC also showed significant OS improvement with the combination of DC-CIK and standard late-line chemotherapy. All these studies demonstrated that DC-based immunotherapy may be combined with systemic chemotherapy to elicit potent systemic antitumor activity and prolong patient survival. In contrast, one phase II randomized clinical trial (9) comparing DCV monotherapy with best supportive care in pretreated patients with mCRC indicated that DCV did not improve PFS and OS, though it can generate the tumor-specific immune response. Therefore, monotherapy by DC-CIK is not supported in patients with advanced mCRC.

In the subgroup analysis, our study found that patients with liver metastasis or ERLN metastasis treated with the combination of DC-CIK and chemotherapy had the dominant survival benefit, compared to chemotherapy alone. In contrast, for patients with peritoneal or lung metastasis, the addition of immunotherapy did not prolong OS, even though cytoreductive surgery with hyperthermia intraperitoneal chemotherapy was performed in four patients (26). This interesting finding can be explained by adoptive T-cell distribution after injection. According to *in vivo* bio-distribution studies, using T-cell imaging tracers, such as ^99^mTc-sum IL-2 (27) or ^111^In-oxine-labeled lymphocyte (28), the dynamic infiltration of adoptive T cells can be investigated, and these studies showed significantly increased uptake of T-cell tracers in some immune organs, including liver, spleen, and tumor-draining lymph node. Therefore, once tumor metastases are detected in these specific organs, the cluster of adoptively transferred T cells can result in a more immune reaction. Current compelling evidence also recommends that the robust intratumoral T-lymphocyte infiltration is a critical part of successful immunotherapy (29, 30).

Another subgroup analysis of this study also demonstrated longer OS by combining DC-CIK with late-line chemotherapy for patients with both RAS and BRAF wild type. In contrast, for patients with refractory mCRC with RAS or BRAF mutation, the combination with immunotherapy did not show any survival advantage. It can be explained by the tumor-intrinsic immune resistance theory in oncogenic RAS signaling. The activation of RAS/MAPK signaling can lead to tumor immune escape and has been correlated with a reduced number of tumor-infiltrating lymphocytes (31). Recognition of cancer cells and response of antitumor T lymphocytes are also dysregulated by oncogenic RAS/MAPK signaling (31). Another study also indicated that the mutant RAS oncoprotein plays a role in upregulating PD-L1 expression, which causes tumor cells to evade the host immune system and enhances immune escape (32). Therefore, the mutant RAS status limited the efficiency of immunotherapy, and DC-CIK was recommended in patients with RAS/BRAF wild type.

In our study, the survival benefit of DC-CIK immunotherapy was consistent in patients with a lower ECOG score. Recent studies have shown that immunotherapy, either alone or in combination with other anticancer treatments, is associated with improved survival irrespective of ECOG 0 (33, 34). The ECOG PS scale is used to determine the ability of a patient to tolerate therapies in serious illness or the potential effect of immunotherapy (35). Patients with NSCLC with poor ECOG PS treated with immune checkpoint inhibitors had significantly worse survival outcomes and were significantly more likely to use healthcare resources (34). Possible reasons for this are as follows: (1) higher immune function—patients with a lower ECOG score may have a stronger immune system, which could enhance the effectiveness of immunotherapy (33); (2) lower tumor burden—patients with a lower ECOG score may have a reduced tumor burden, which could make them more responsive to immunotherapy (36); and (3) better treatment adherence—patients with a lower ECOG score are more likely to adhere to treatment regimens, which could improve treatment outcomes (37). It is important to note that the relationship between ECOG score and immunotherapy outcomes is complex and further research is needed.

Although we used case–control methodology to match patient characteristics, our retrospective study still had some limitations. First, selection bias could not be completely eliminated. Because this is a real-world retrospective clinical study, the socioeconomic bias between two groups is difficult to ignore from the high cost of ACT immunotherapy. Second, in Taiwan, cell therapy has been approved for cancer therapy since June 2019, but Regorafenib has been used since April 2015. Therefore, the treatment period between the cases and controls reveals a 4-year gap. Third, our results showed the trend of PFS benefit in DC-CIK cases, but no statistically significant difference (*p* = 0.086), which was associated with higher tumor volume burden and having more patients with peritoneal metastasis in the DC-CIK case group. Finally, our sample size for DC-CIK cases is too small and the follow-up time is too short for further analysis. Accordingly, a randomized control study with a large population is needed to substantiate our results.

In conclusion, DC-CIK combined with chemotherapy offers patients with refractory mCRC (>2 previous chemotherapy lines) statistically significantly longer OS, especially in the patient subgroup with liver metastasis or ERLN metastasis or those with an ECOG score of 0 or both RAS/BRAF wild type. The findings of this study open new perspectives for future prospective randomized trials to determine the value of ACT immunotherapy in patients with highly pretreated mCRC.

## Data availability statement

The original contributions presented in the study are included in the article/**Supplementary Material**. Further inquiries can be directed to the corresponding author.

## Ethics statement

The studies involving humans were approved by CMUH111-REC3-191, China Medical University Hospital Research Ethics Committee. The studies were conducted in accordance with the local legislation and institutional requirements. The ethics committee/institutional review board waived the requirement of written informed consent for participation from the participants or the participants’ legal guardians/next of kin because this is a retrospective study and data analysis was done from recorded chart.

## Author contributions

S-CC: Conceptualization, Data curation, Formal analysis, Funding acquisition, Investigation, Methodology, Project administration, Resources, Software, Supervision, Validation, Visualization, Writing – original draft, Writing – review & editing. L-BJ: Conceptualization, Formal analysis, Resources, Supervision, Visualization, Writing – review & editing. T-WK: Investigation, Writing – review & editing. W-LC: Conceptualization, Writing – review & editing. W-CS: Funding acquisition, Validation, Writing – review & editing.

## Supplementary material

Supplementary 1 Patient’s allocation algorithm.
