# Supplementary material for: Effect of autologous dendritic cell cytokine-induced killer on refractory metastatic colorectal cancer: a matched case–control comparative study
Source: Front Immunol. 2024 Feb 27;15:1329615. doi: 10.3389/fimmu.2024.1329615 (PMC10927724; doi:10.3389/fimmu.2024.1329615)

Case 1

DC markers

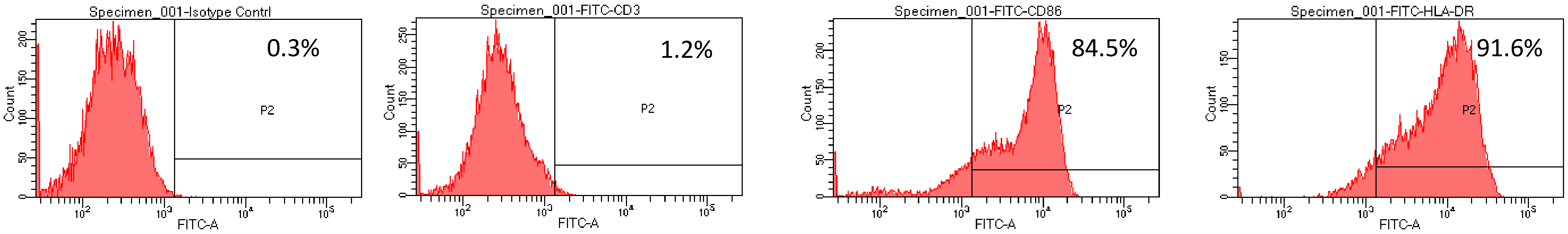

DC-CIK markers

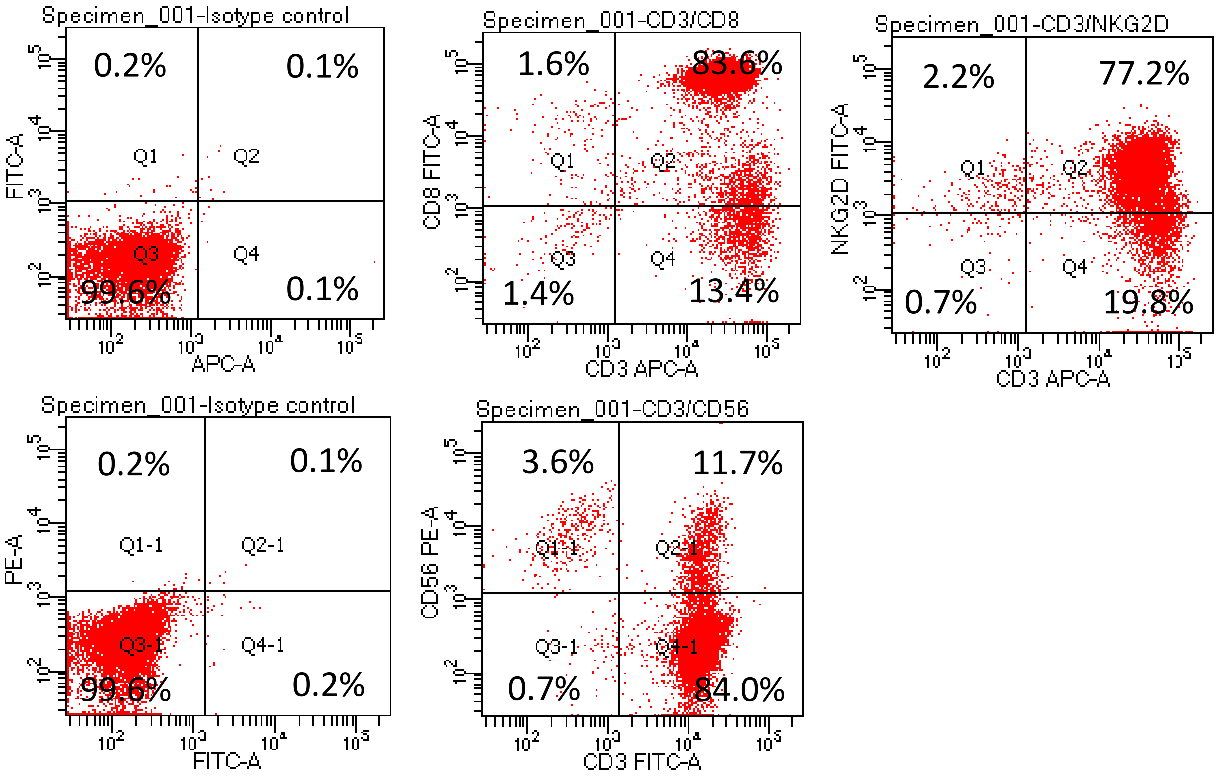

Case 2

DC markers

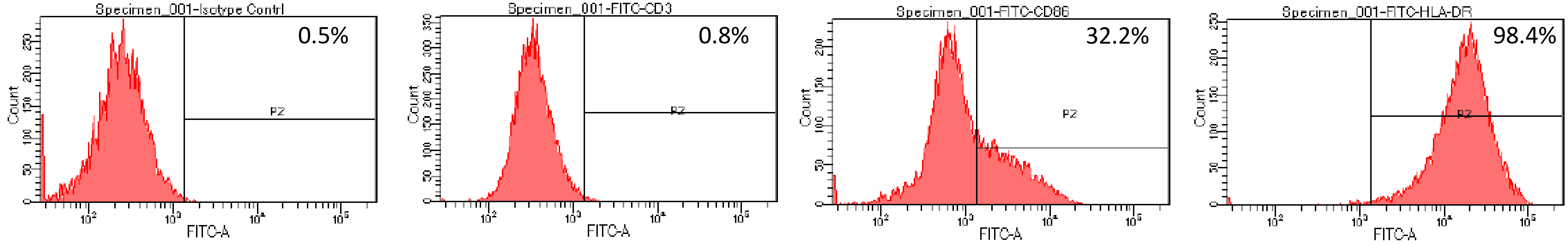

DC-CIK markers

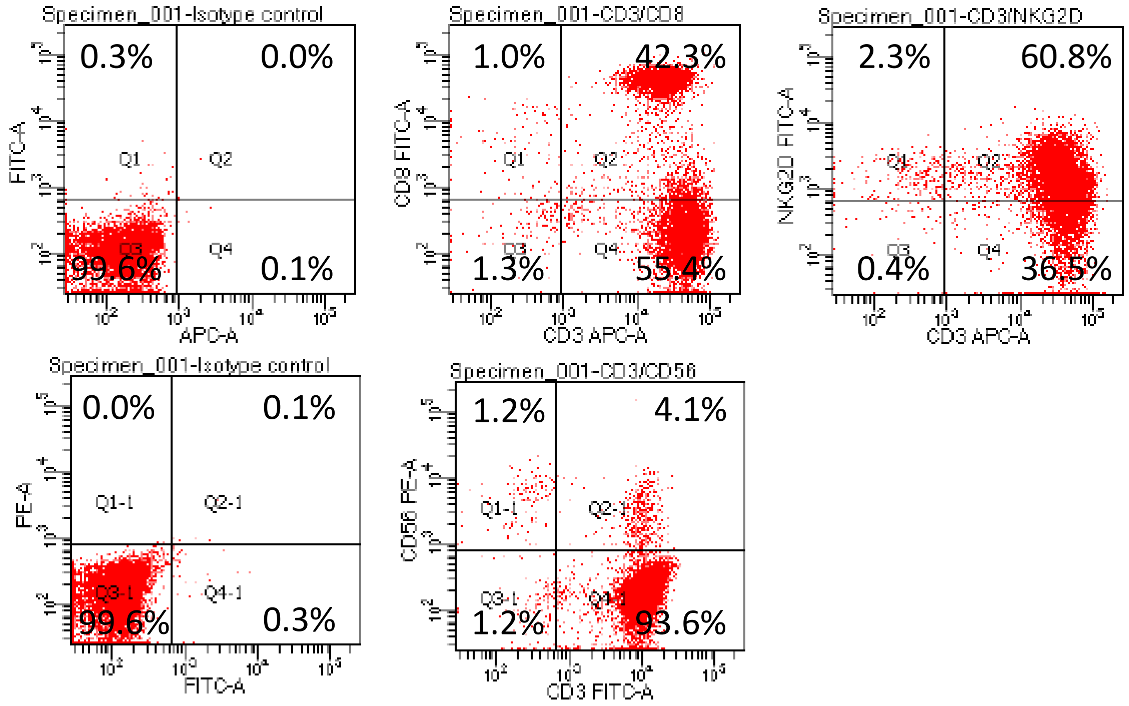

Case 3

DC markers

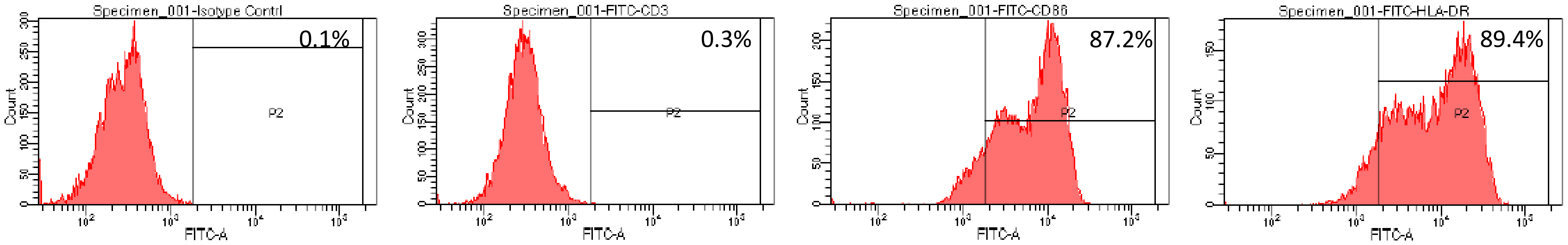

DC-CIK markers

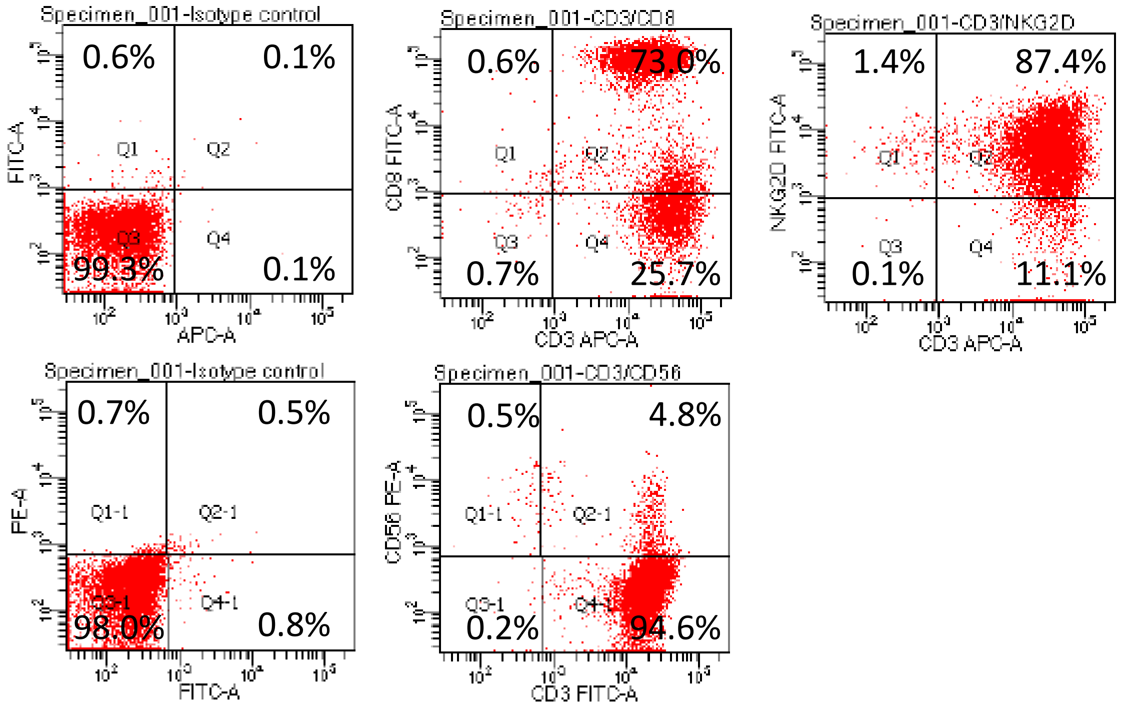

Case 4

DC markers

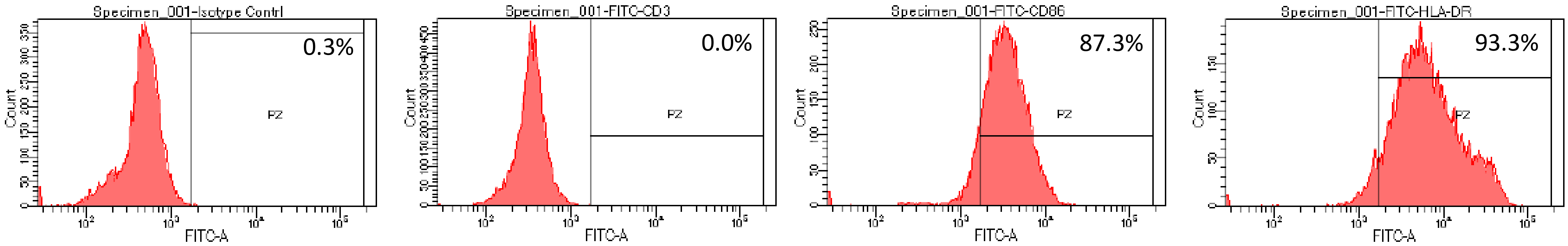

DC-CIK markers

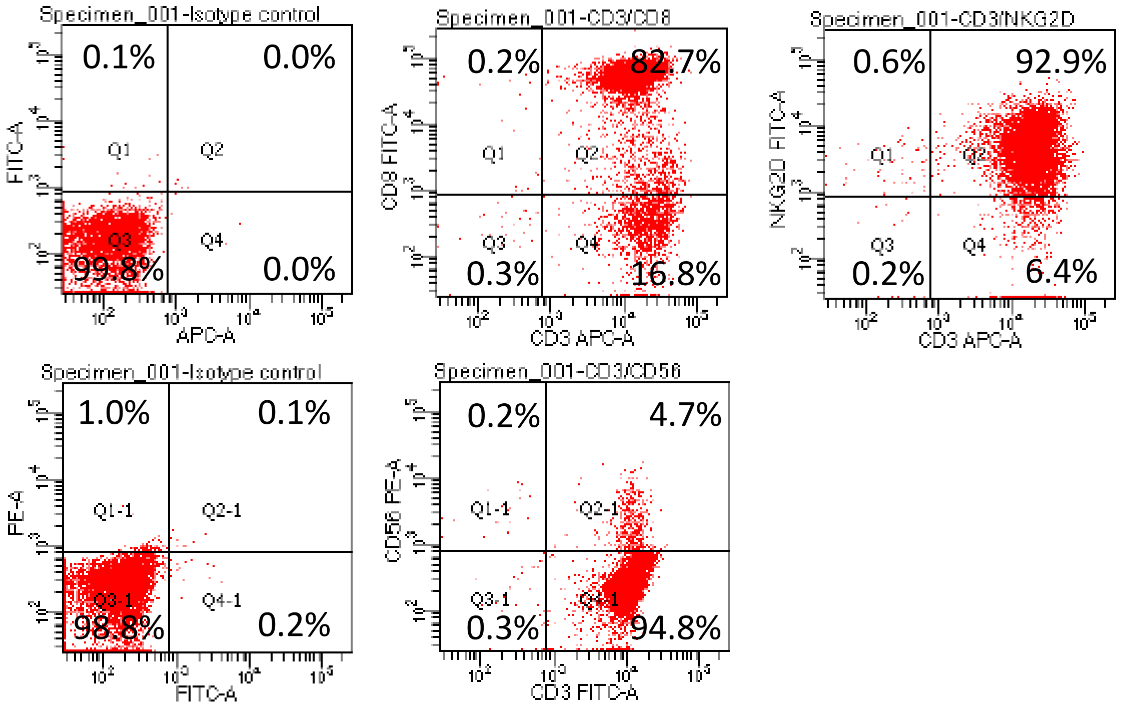

Case 5

DC markers

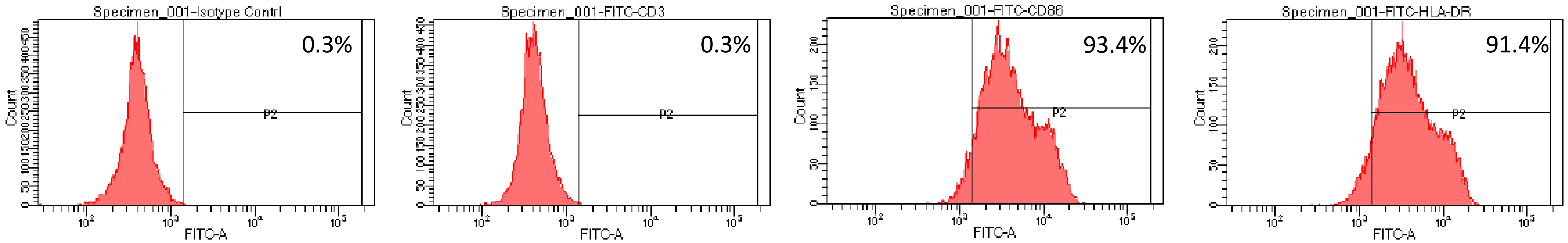

DC-CIK markers

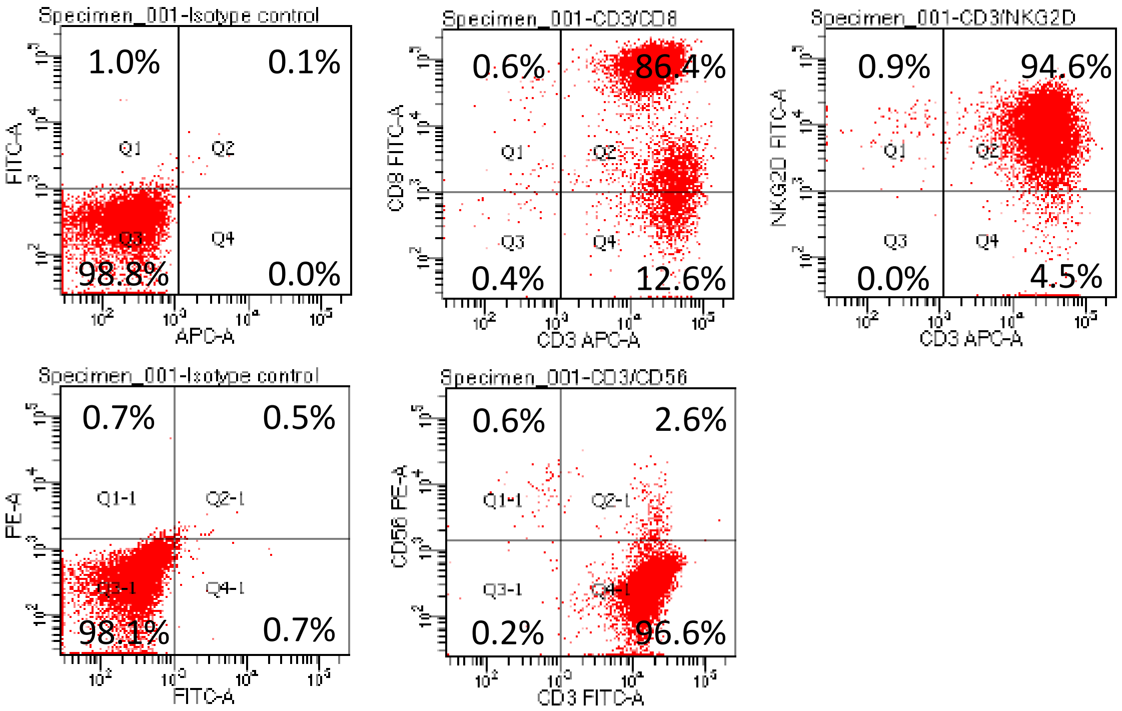

Case 6

DC markers

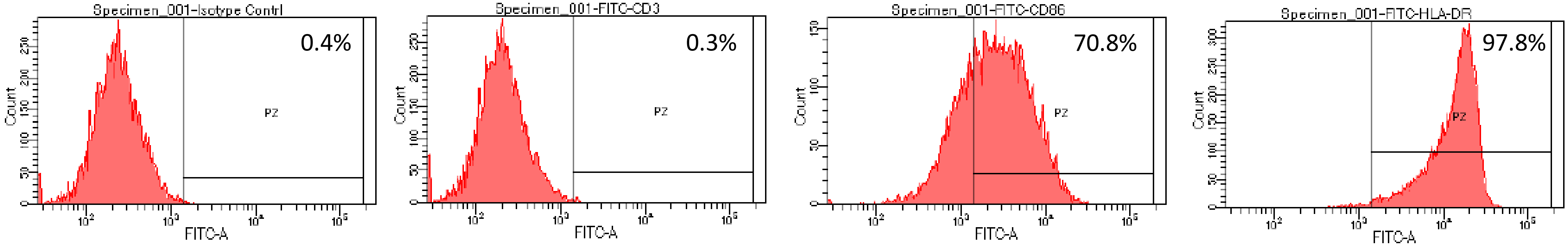

DC-CIK markers

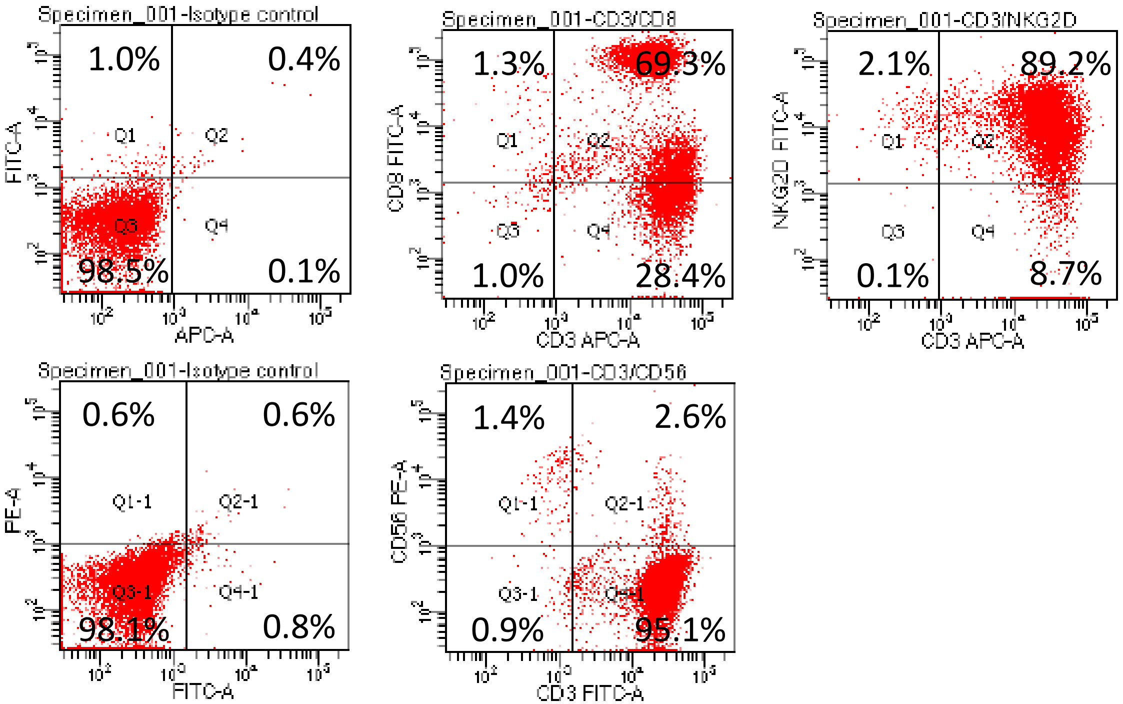

Case 7

DC markers

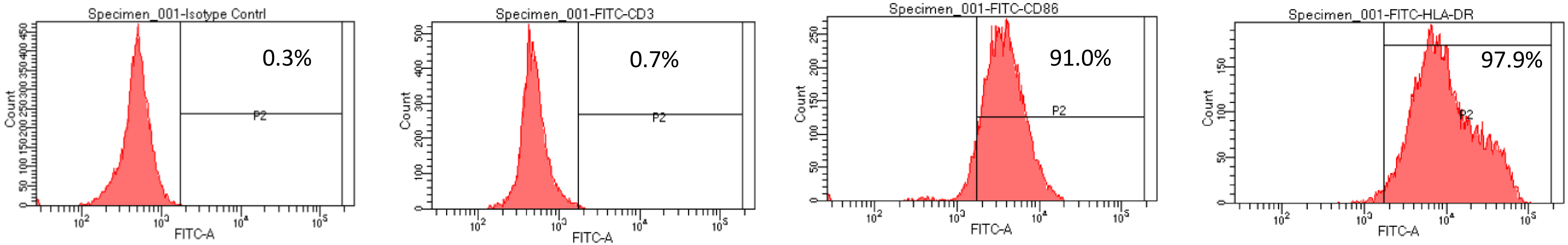

DC-CIK markers

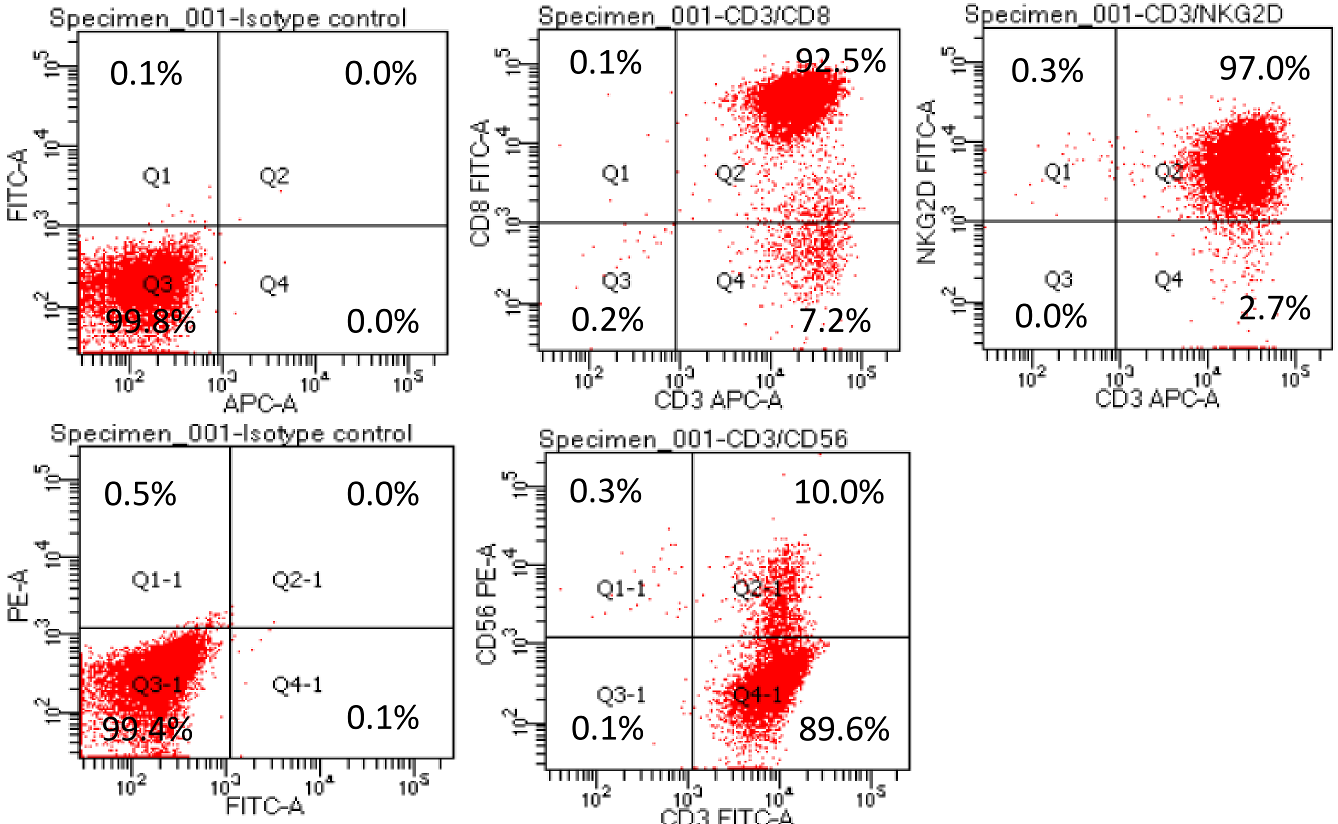

Case 8

DC markers

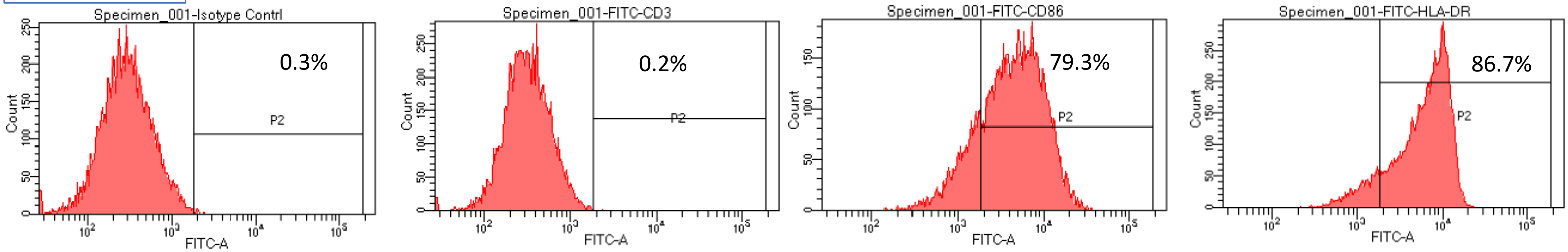

DC-CIK markers

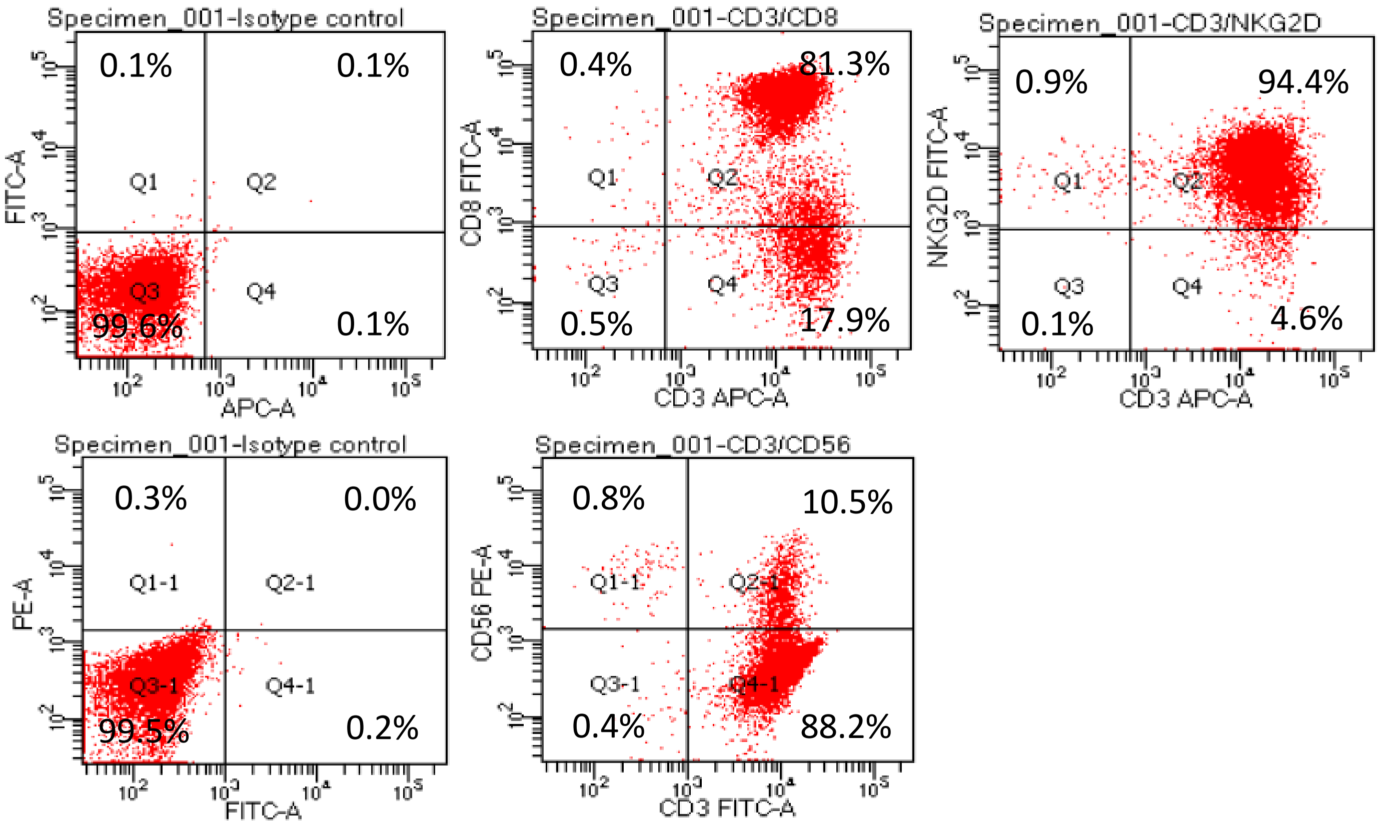

Case 9

DC markers

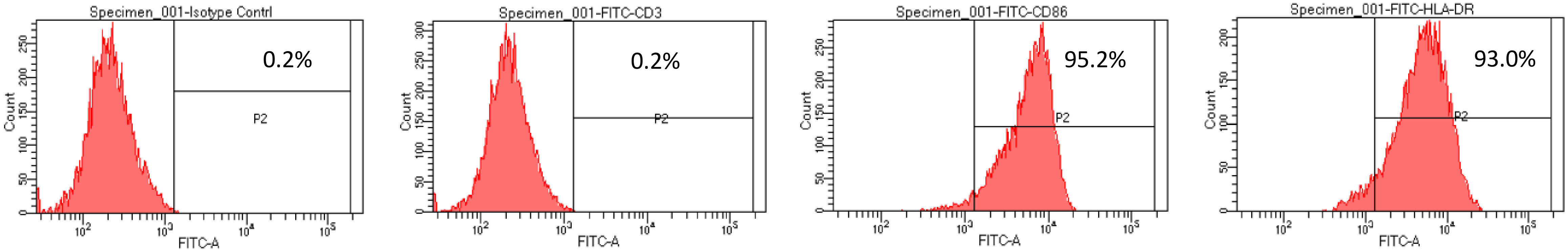

DC-CIK markers

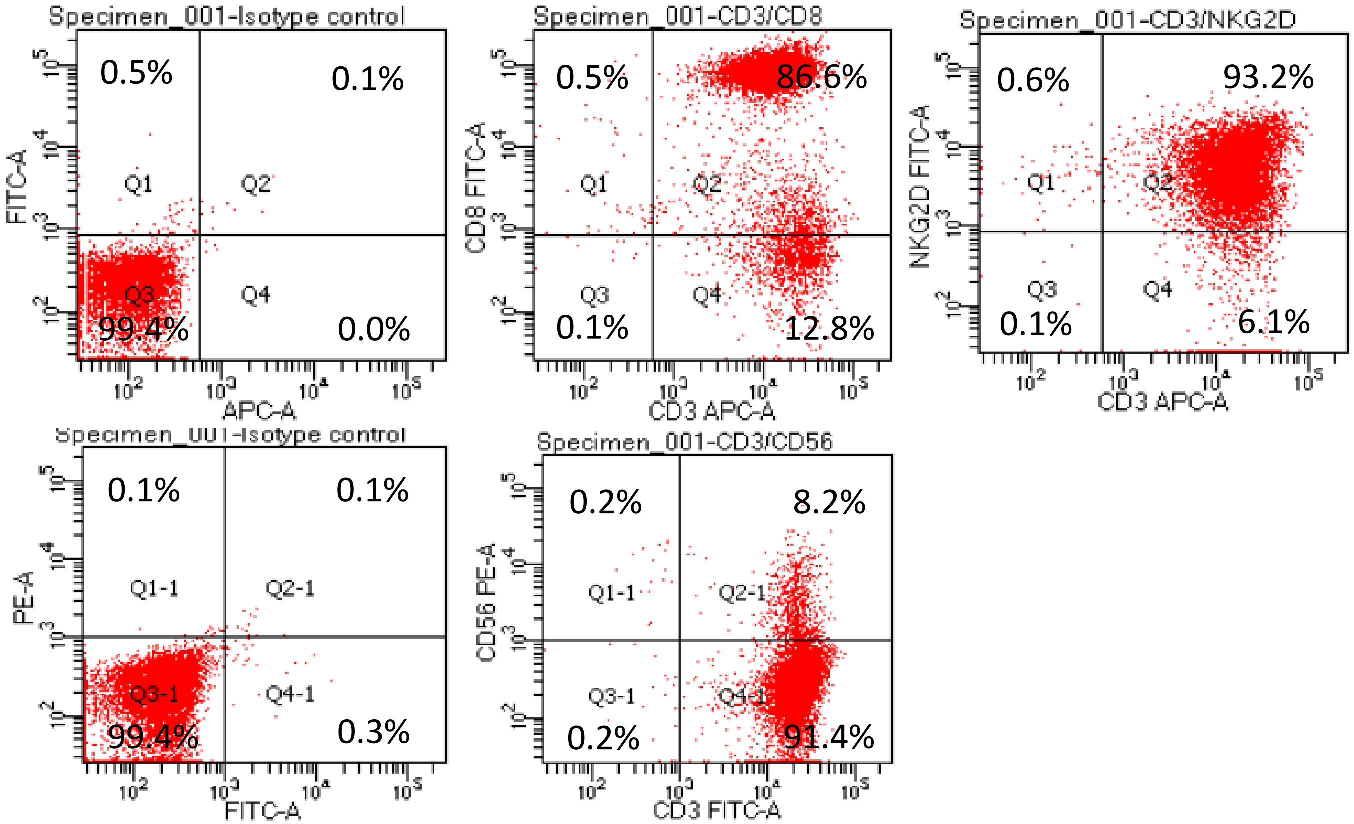

Case 10

DC markers

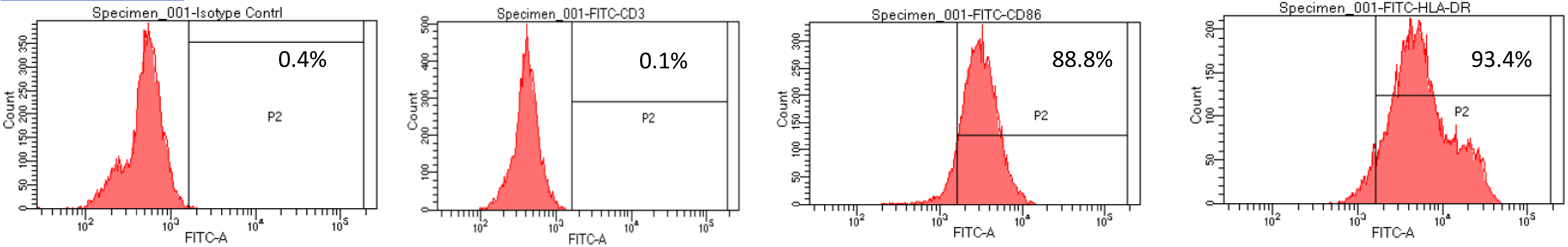

DC-CIK markers

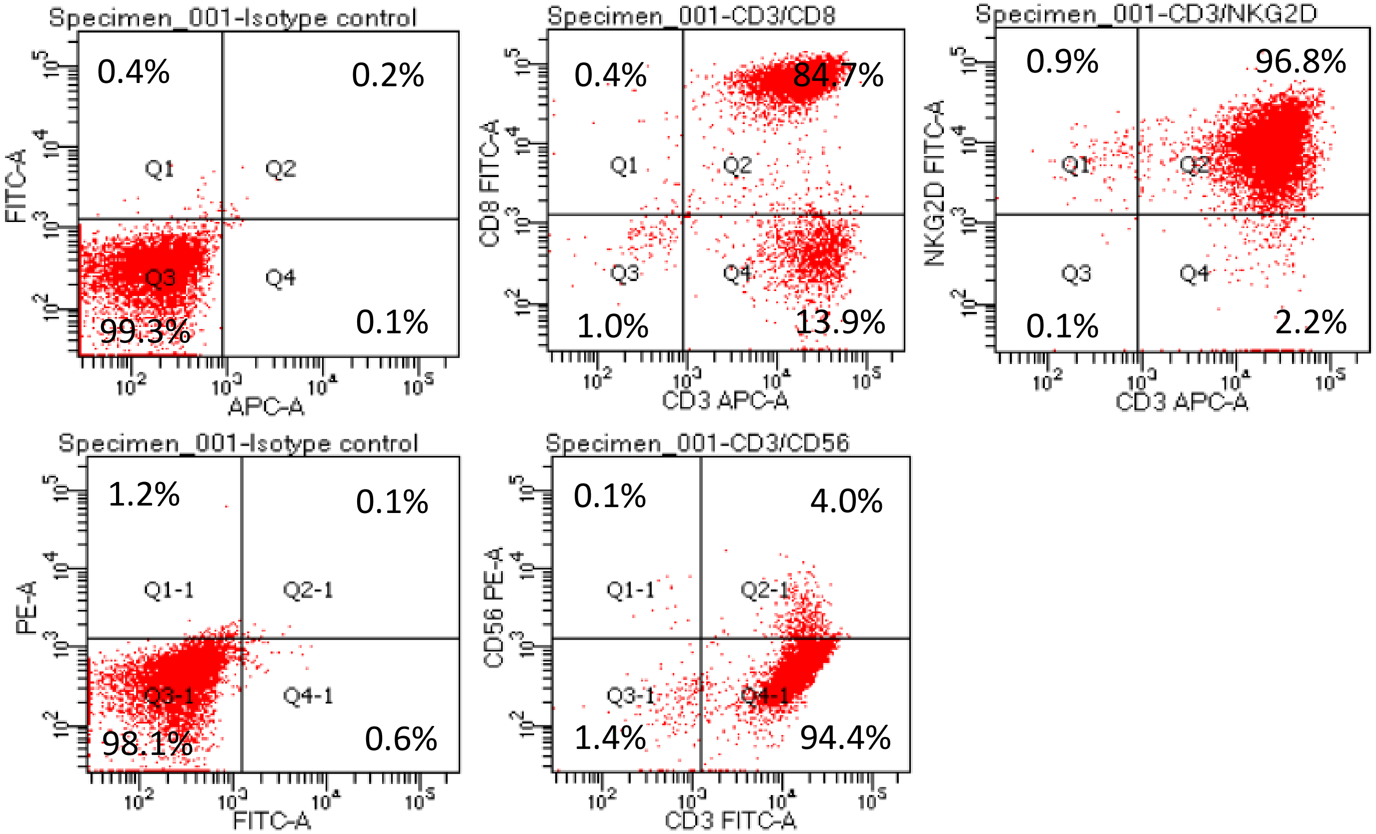

Case 11

DC markers

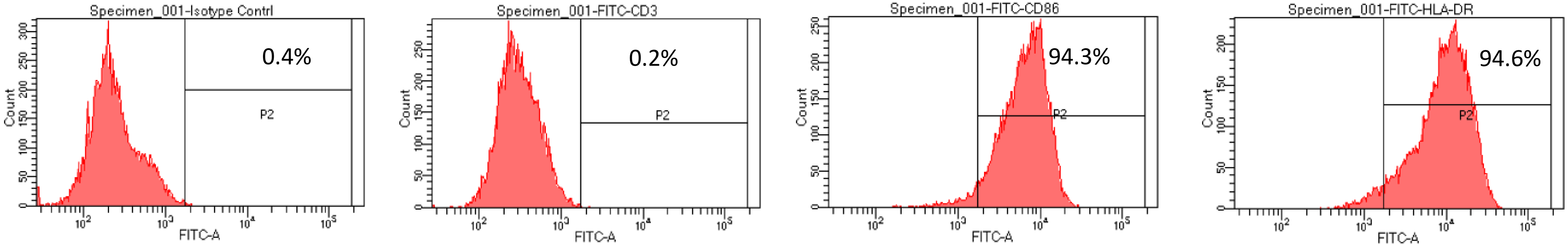

DC-CIK markers

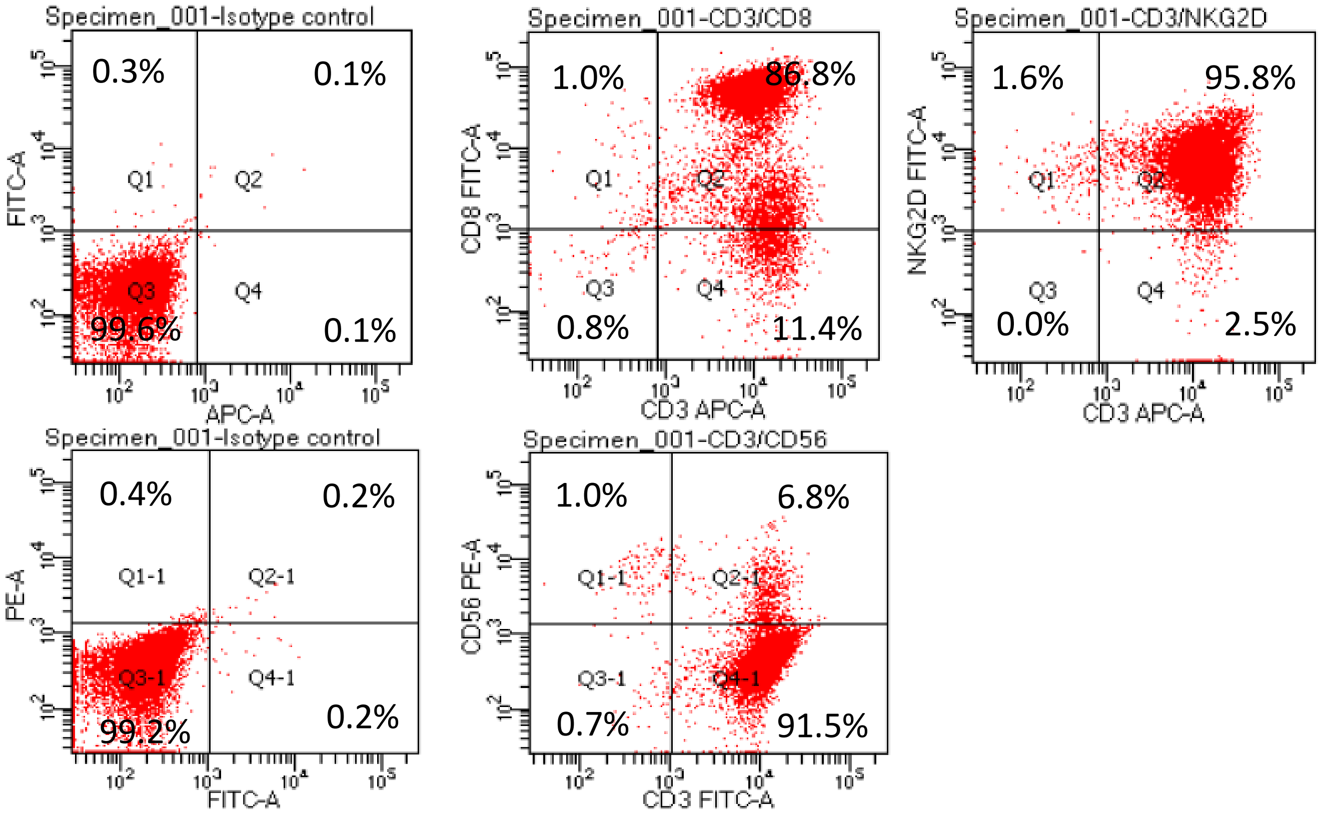

Case 12

DC markers

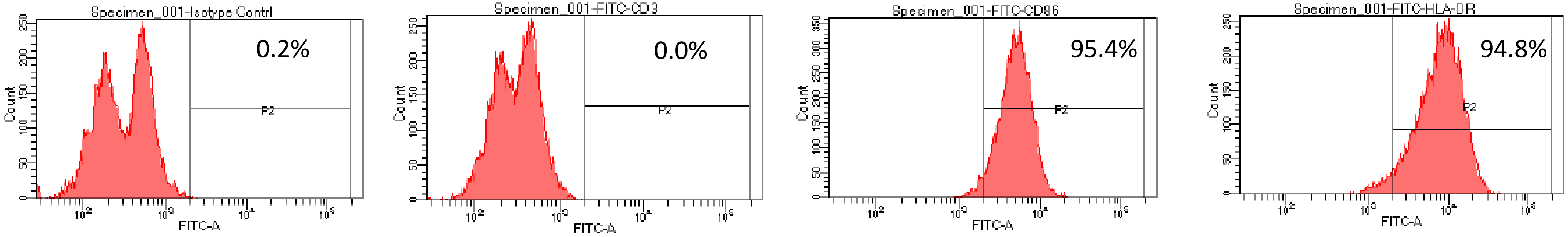

DC-CIK markers

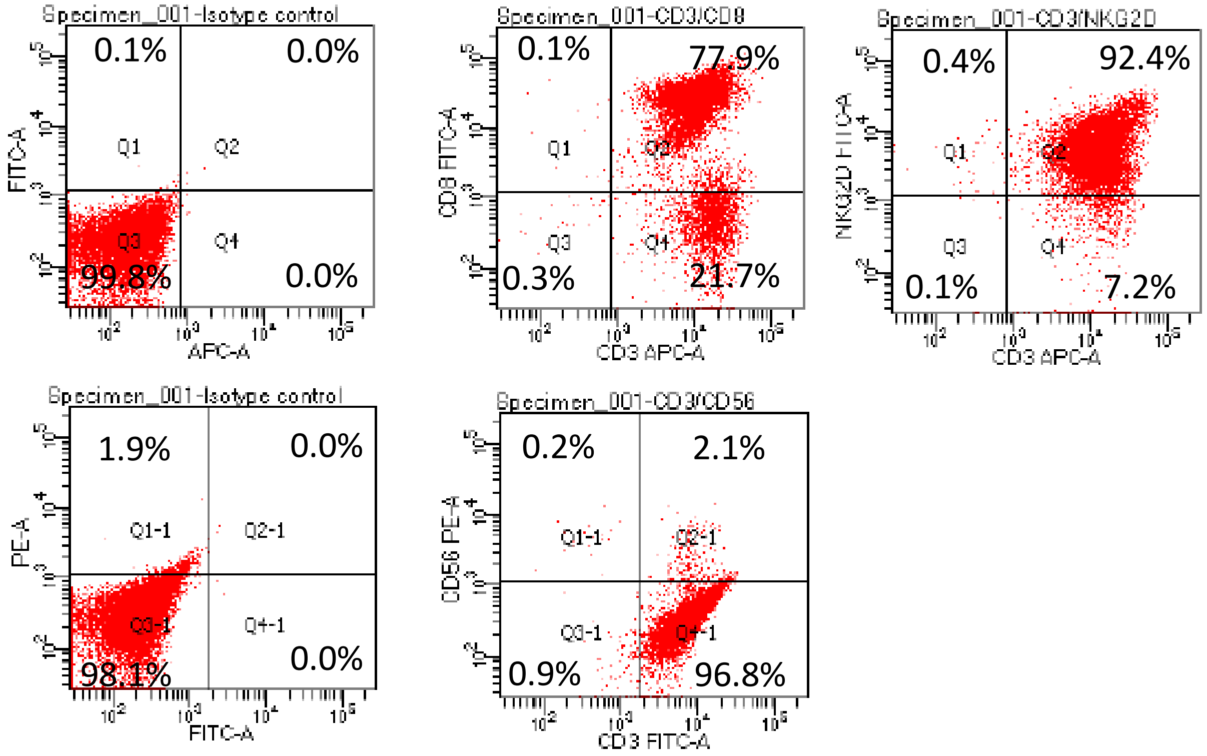

Case 3

DC markers

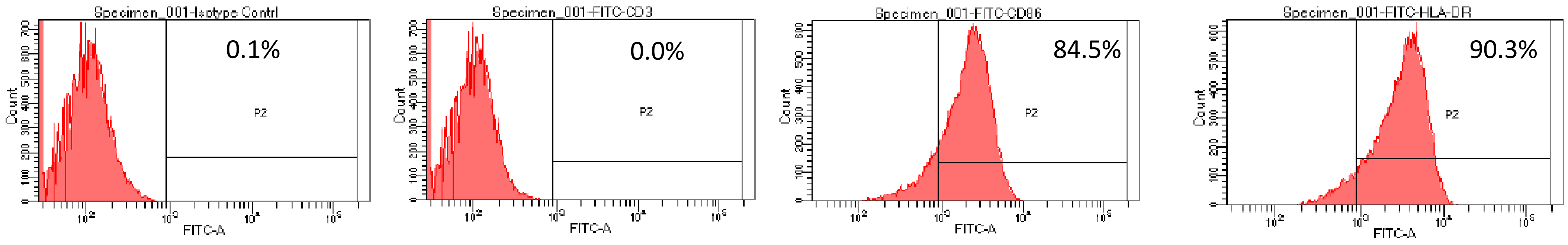

DC-CIK markers

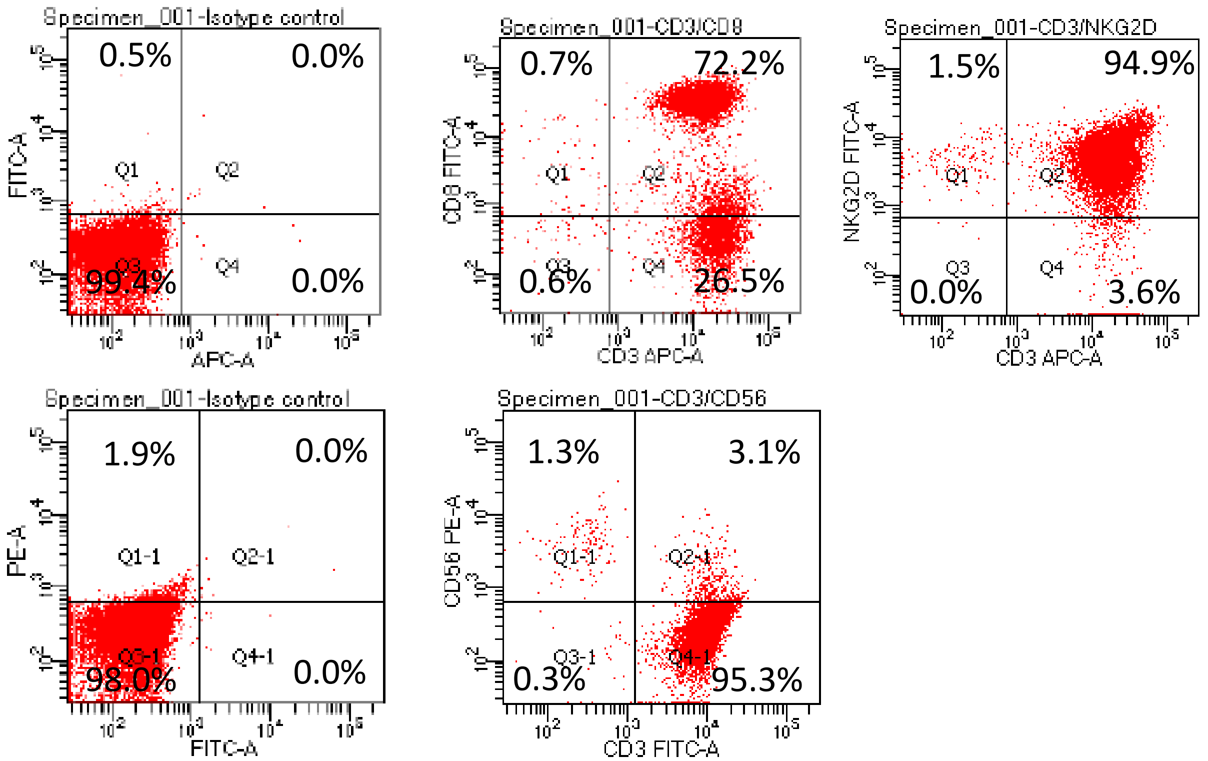

Case 14

DC markers

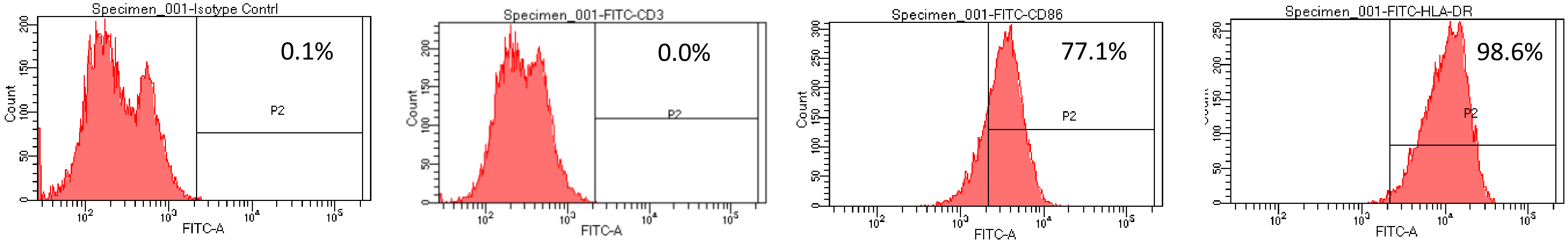

DC-CIK markers

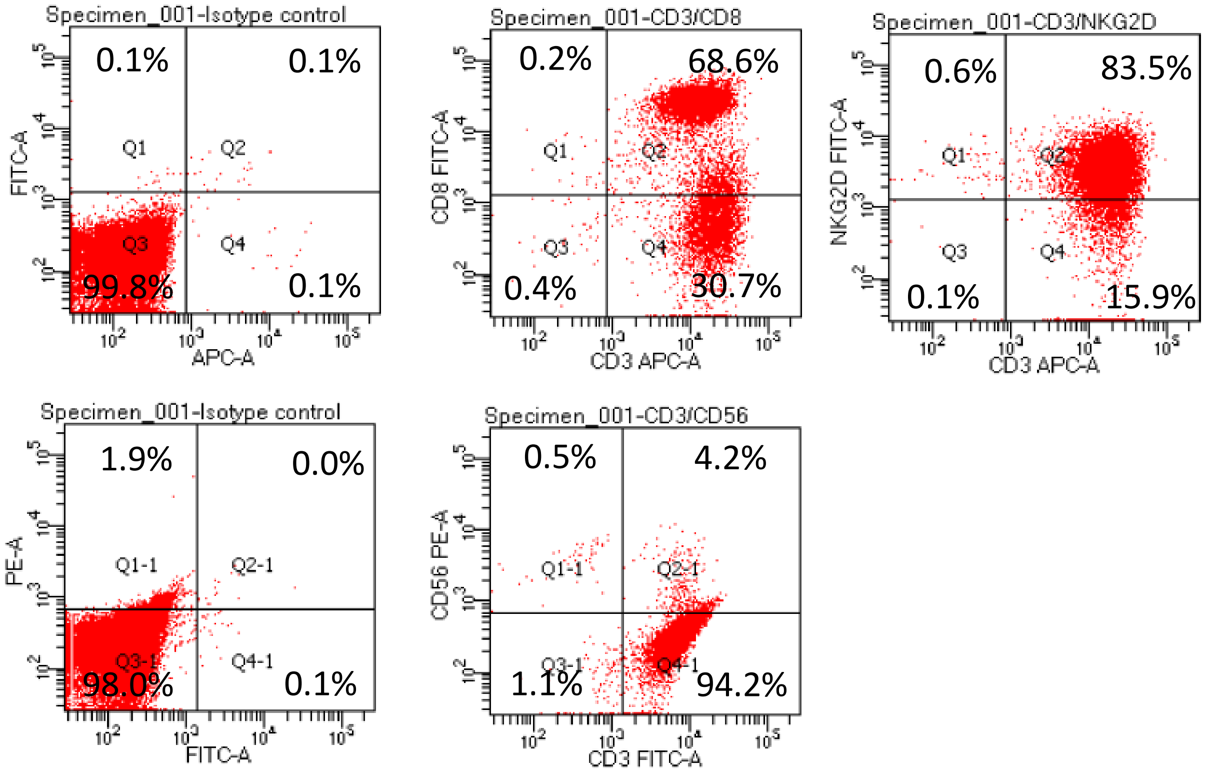

Case 15

DC markers

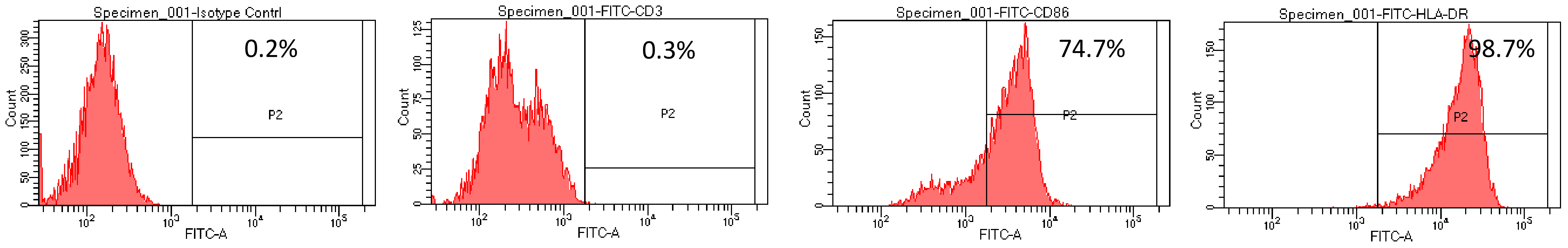

DC-CIK markers

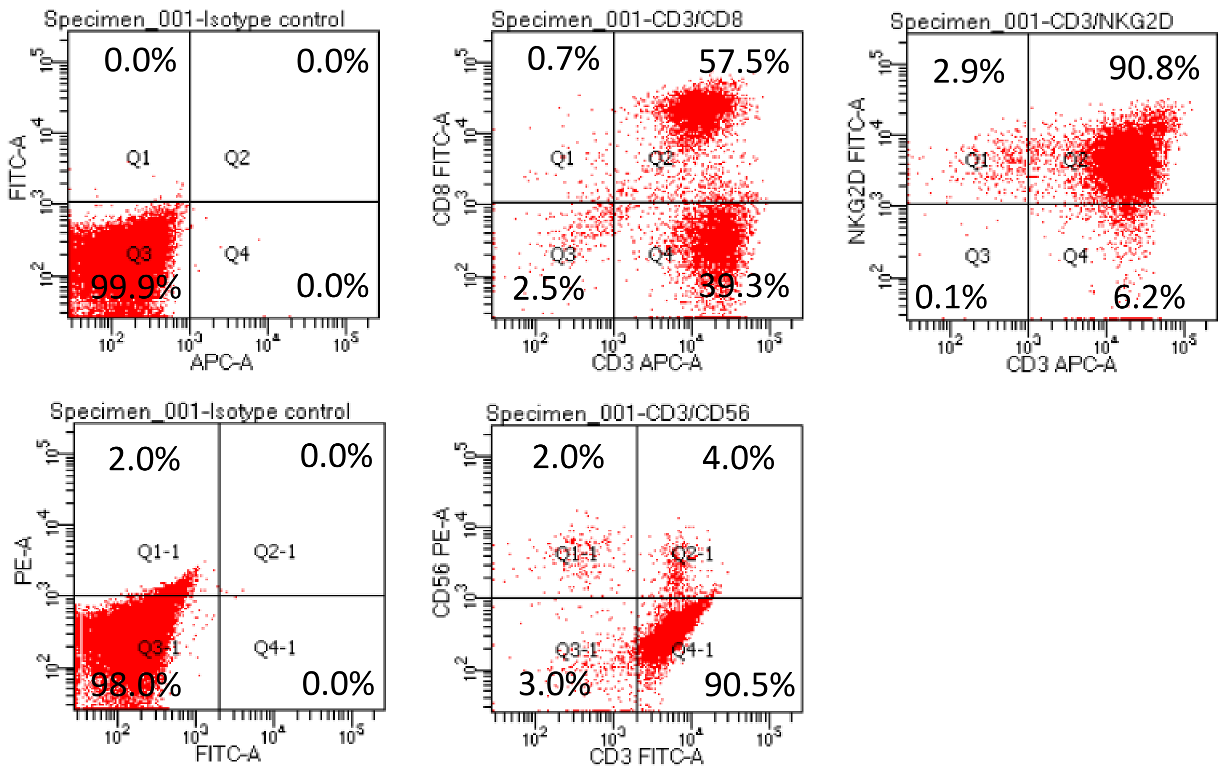

Case 16

DC markers

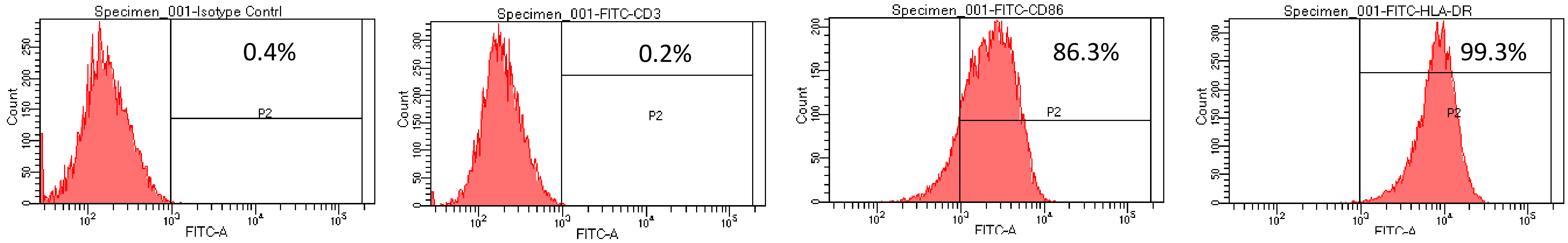

DC-CIK markers

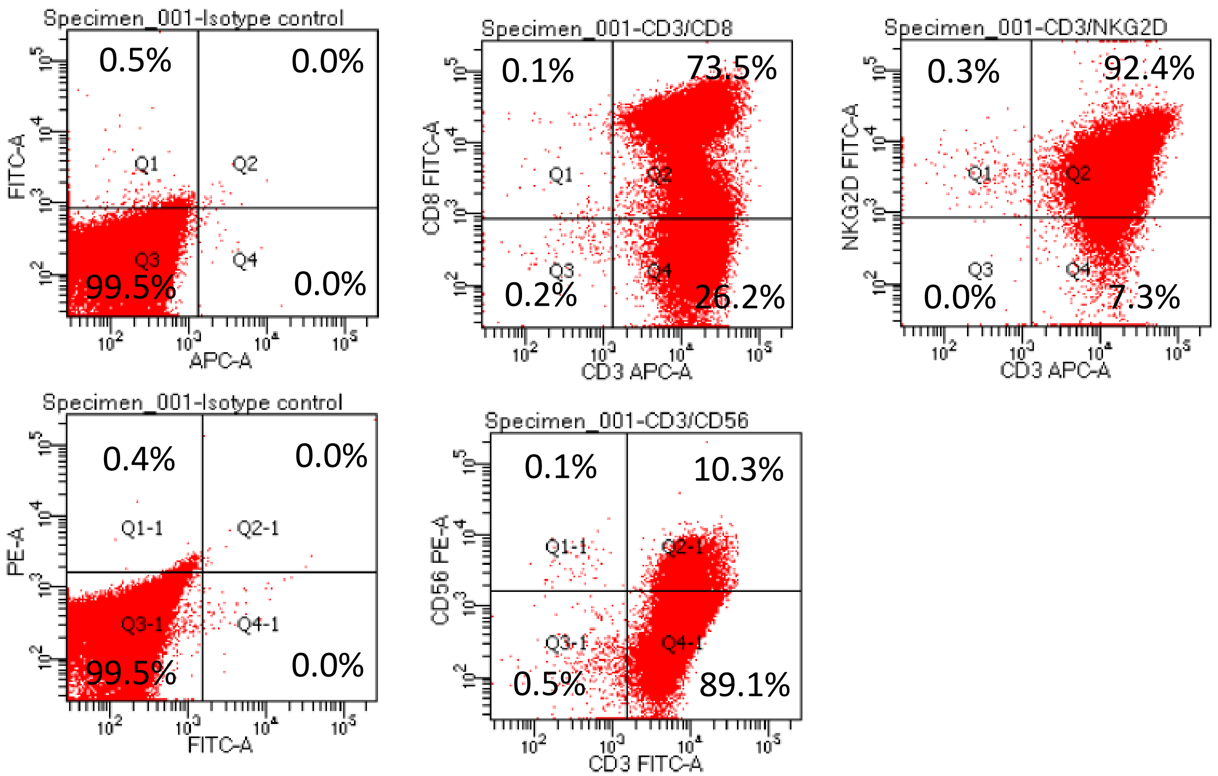

Case 17

DC markers

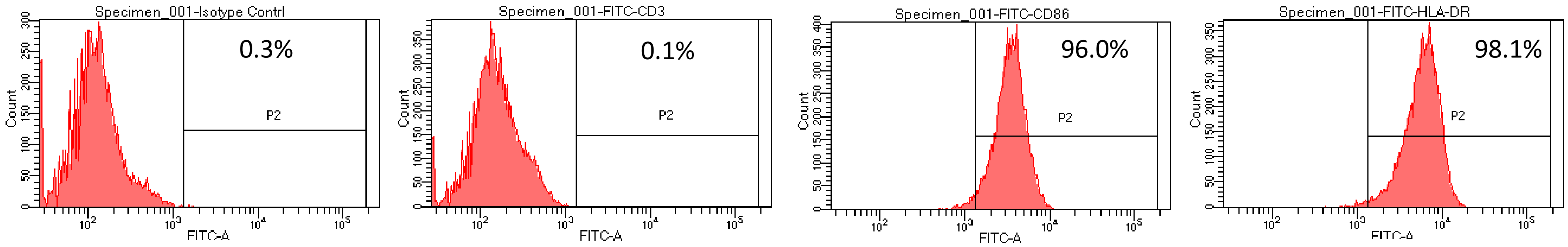

DC-CIK markers

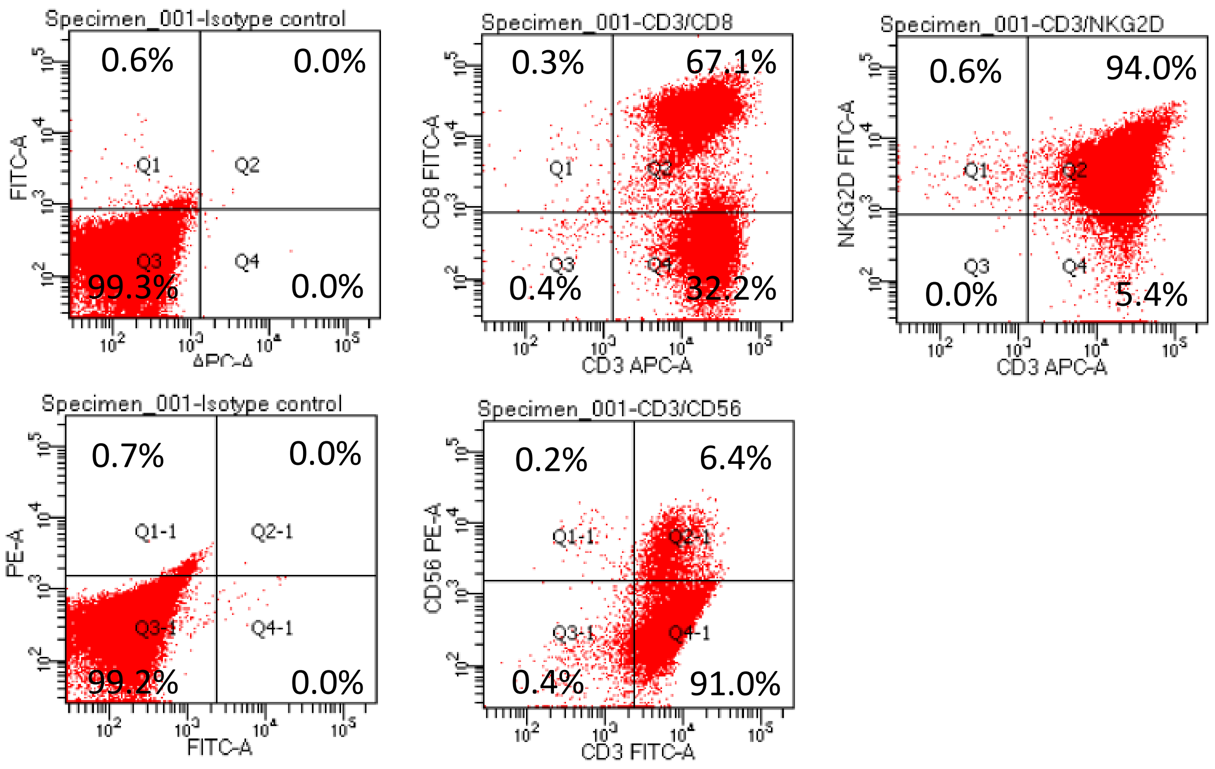

Case 18

DC markers

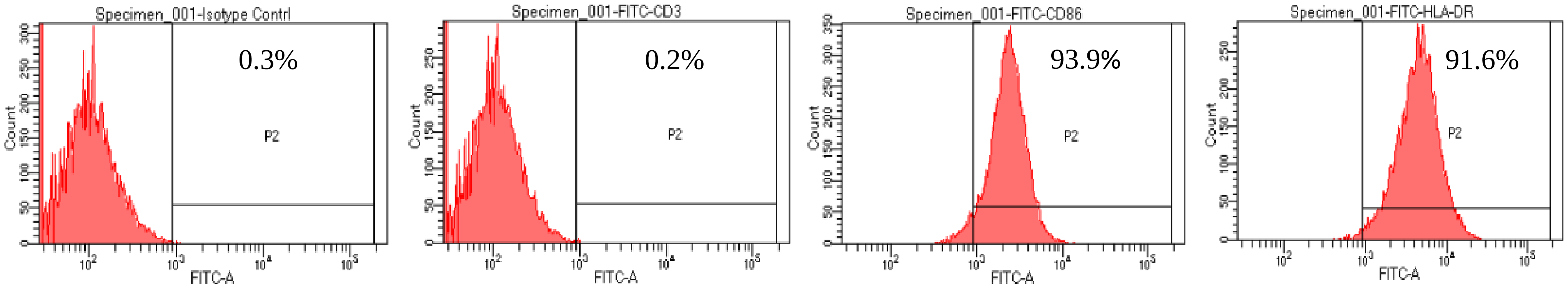

DC-CIK markers

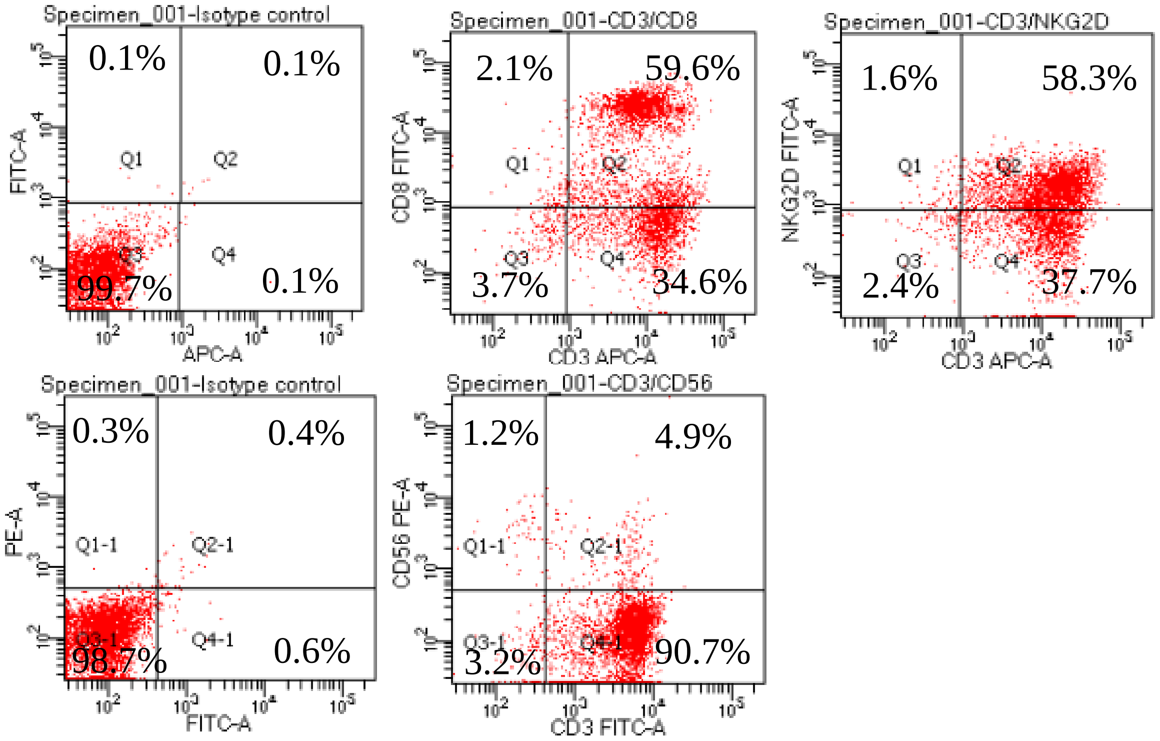

Case 19

DC markers

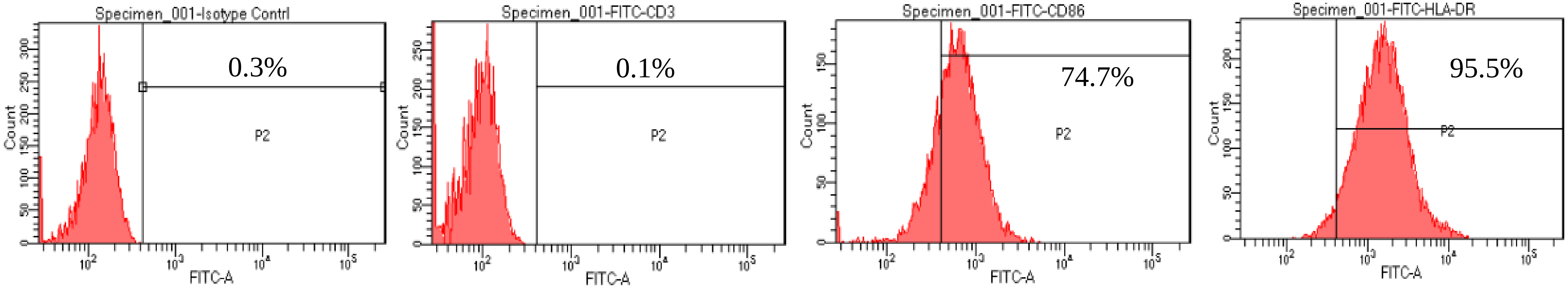

DC-CIK markers

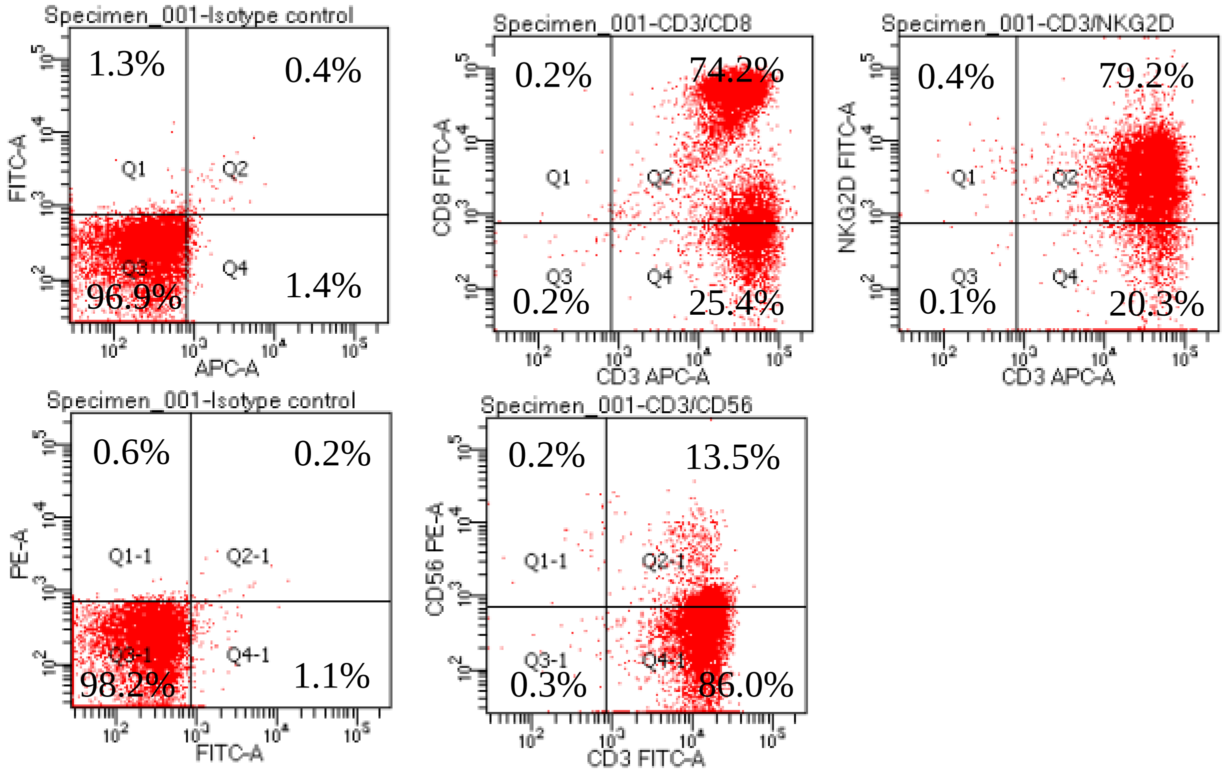

Case 20

DC markers

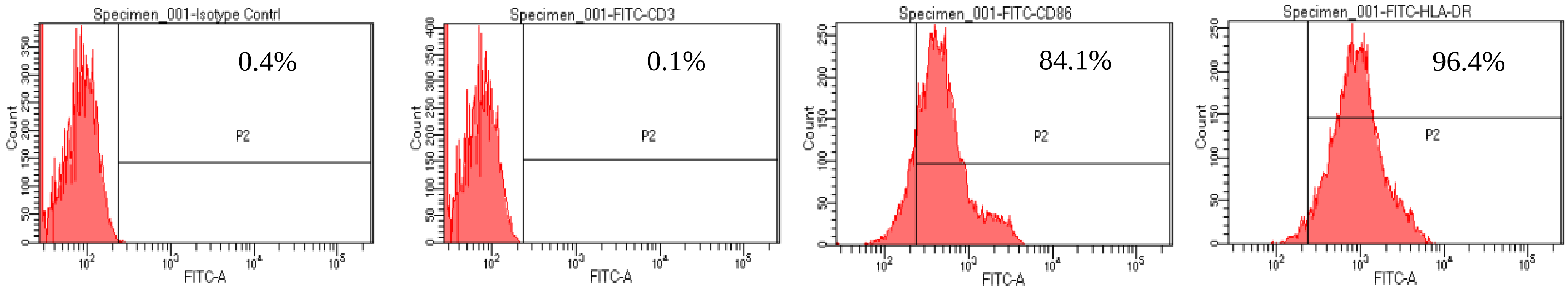

DC-CIK markers

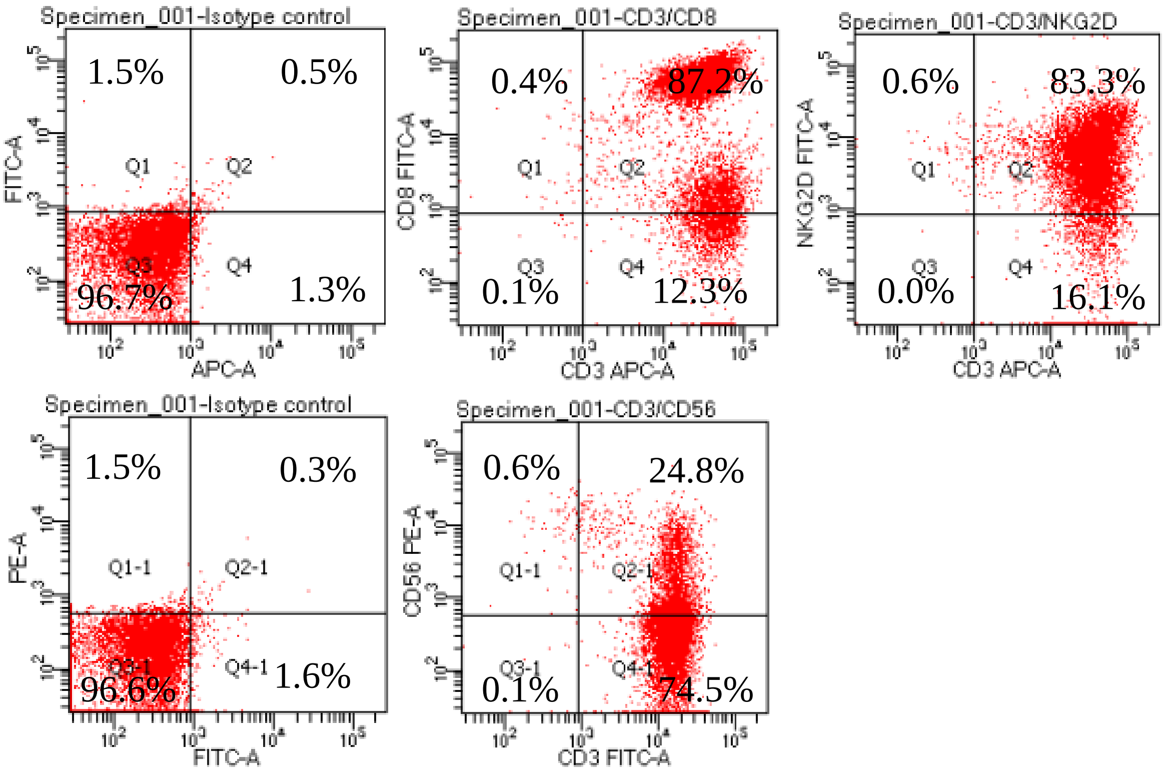

Case 21

DC markers

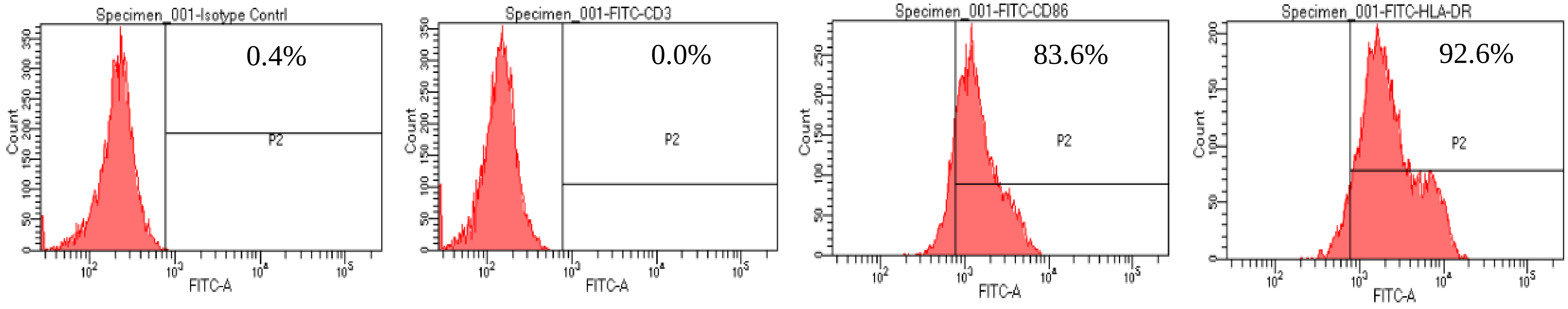

DC-CIK markers

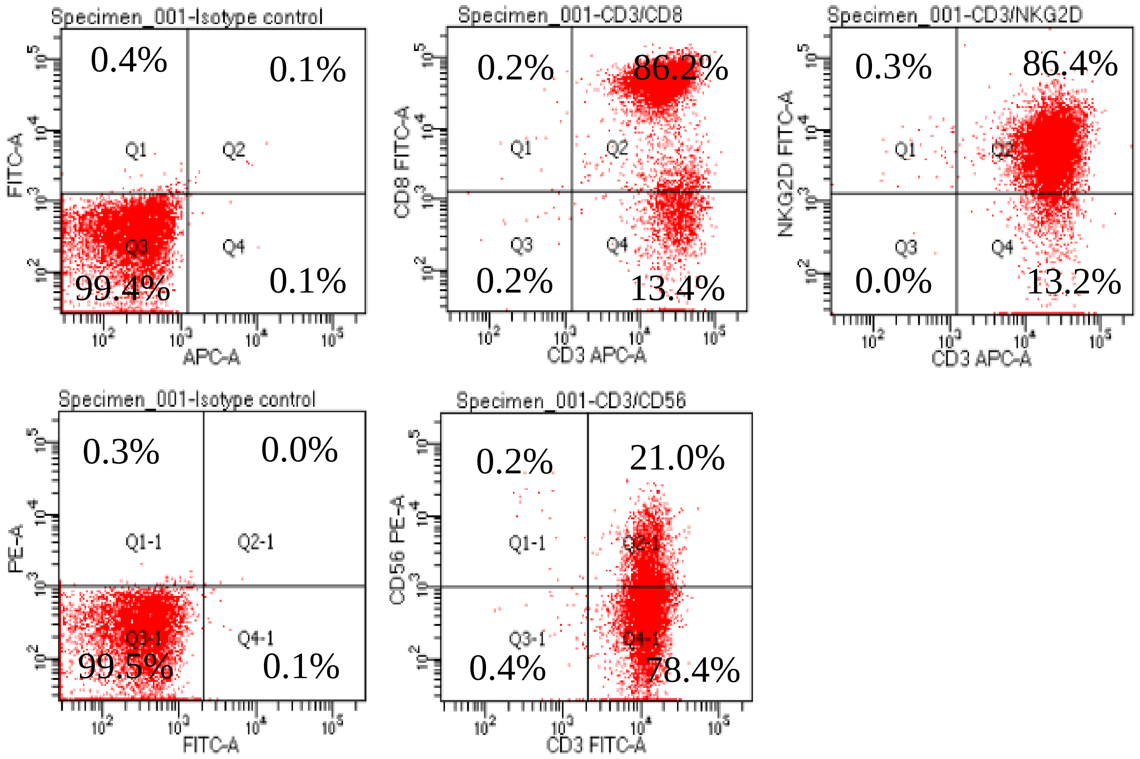

Case 22

DC markers

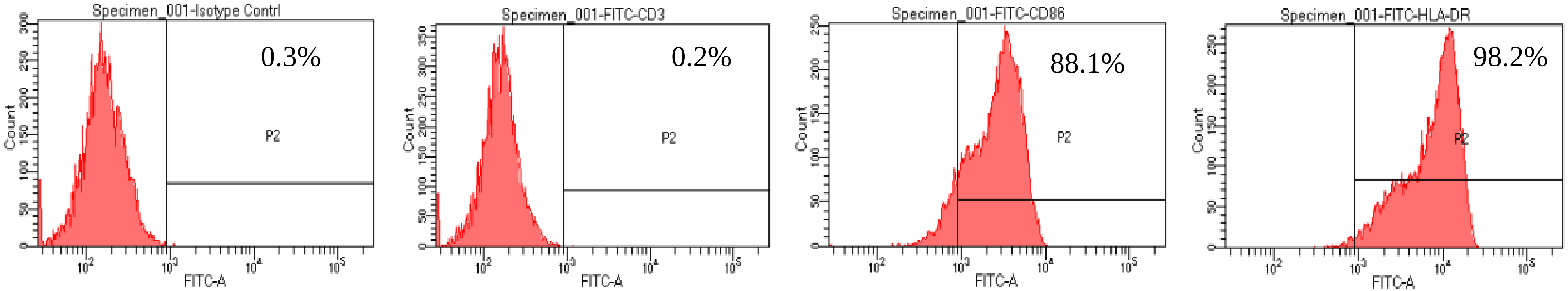

DC-CIK markers

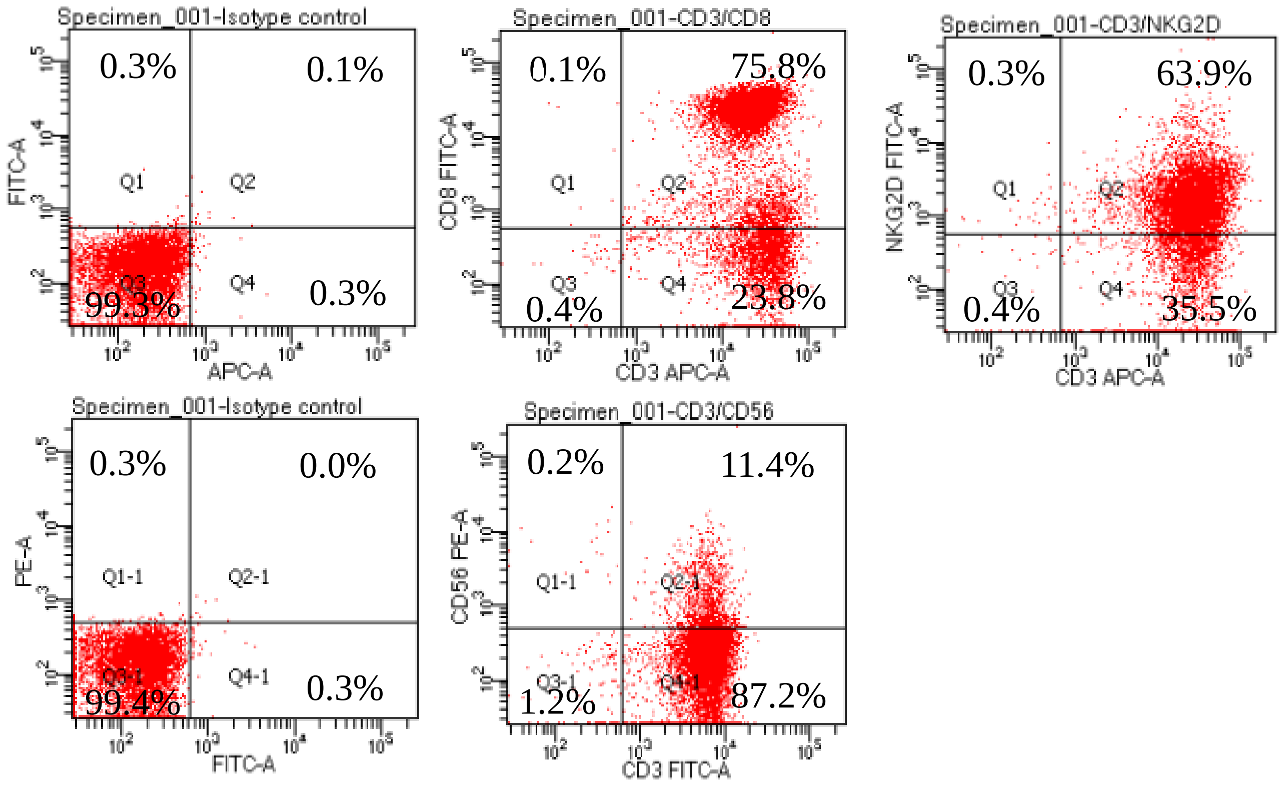

Case 23

DC markers

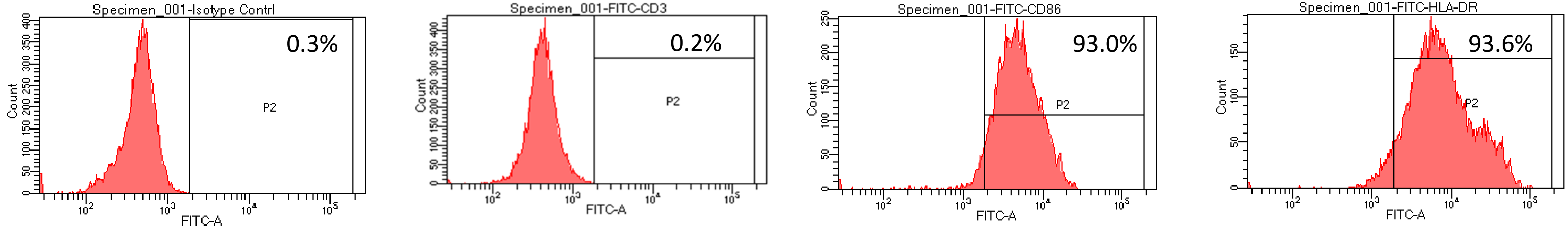

DC-CIK markers

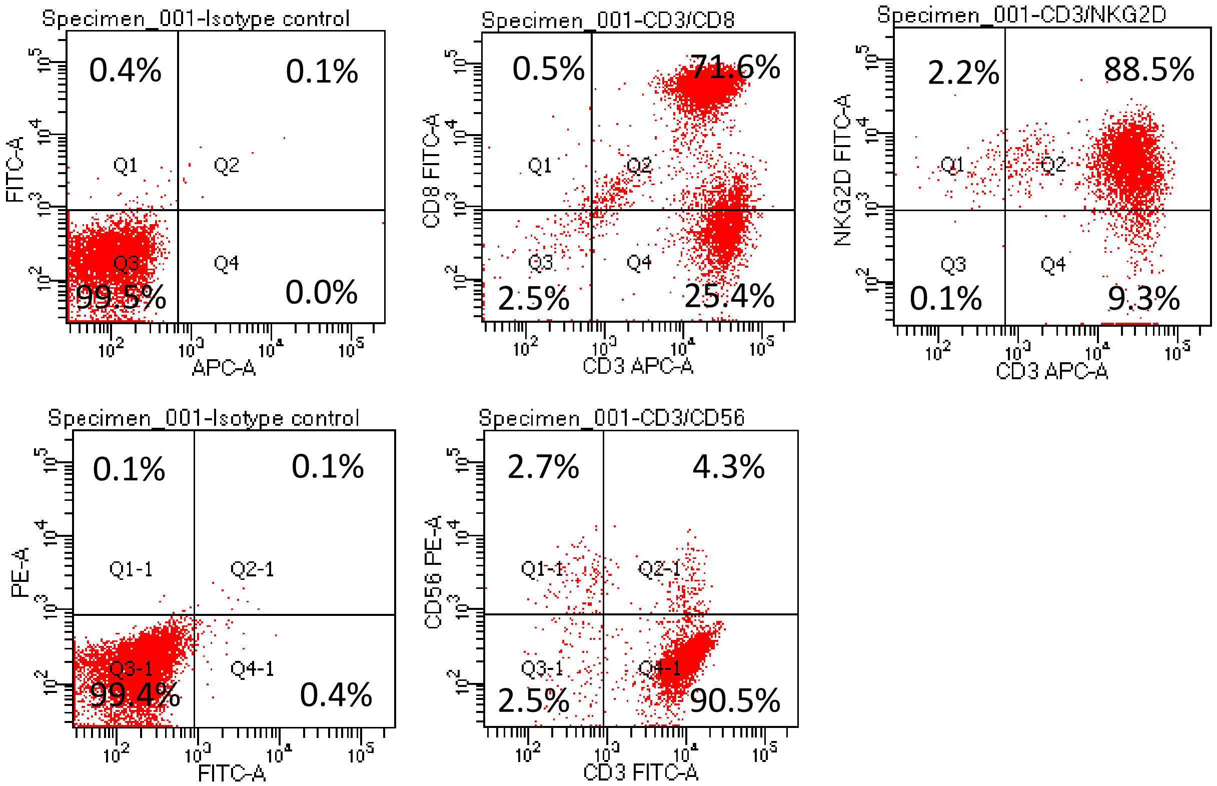

Case 24

DC markers

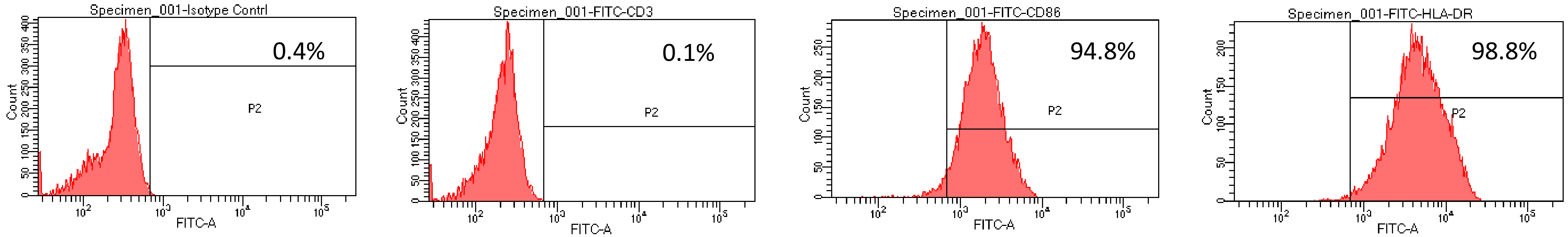

DC-CIK markers

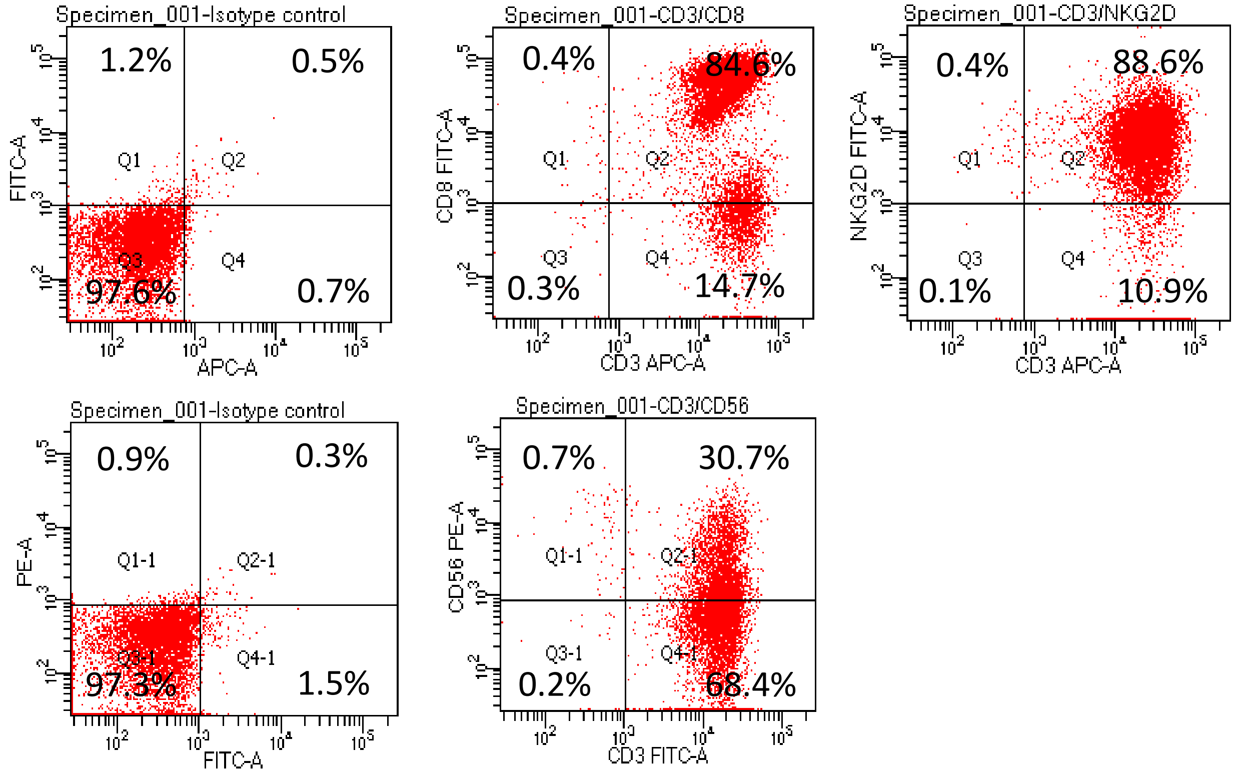

Case 25

DC markers

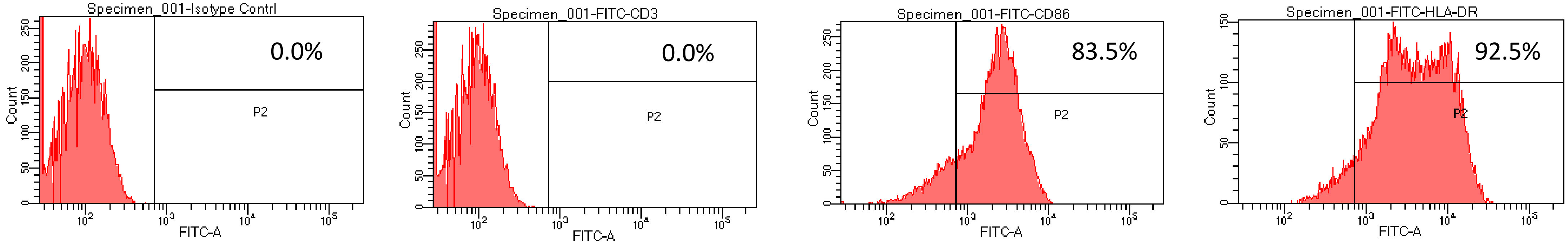

DC-CIK markers

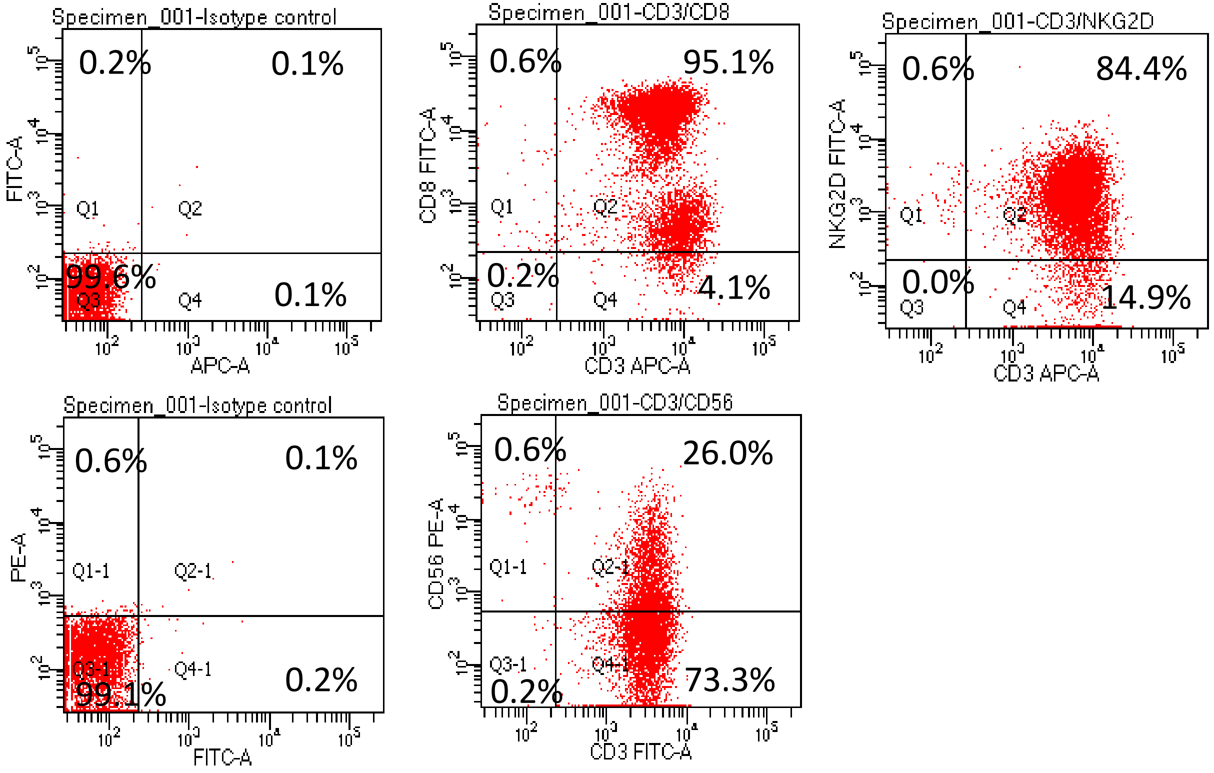

Case 26

DC markers

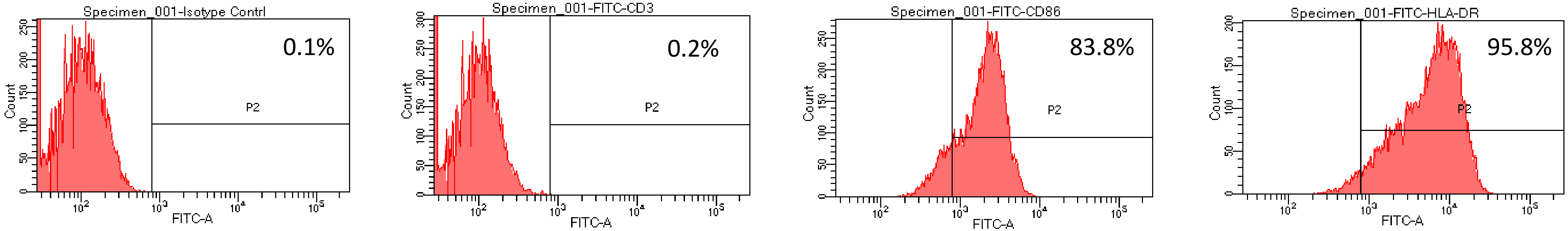

DC-CIK markers

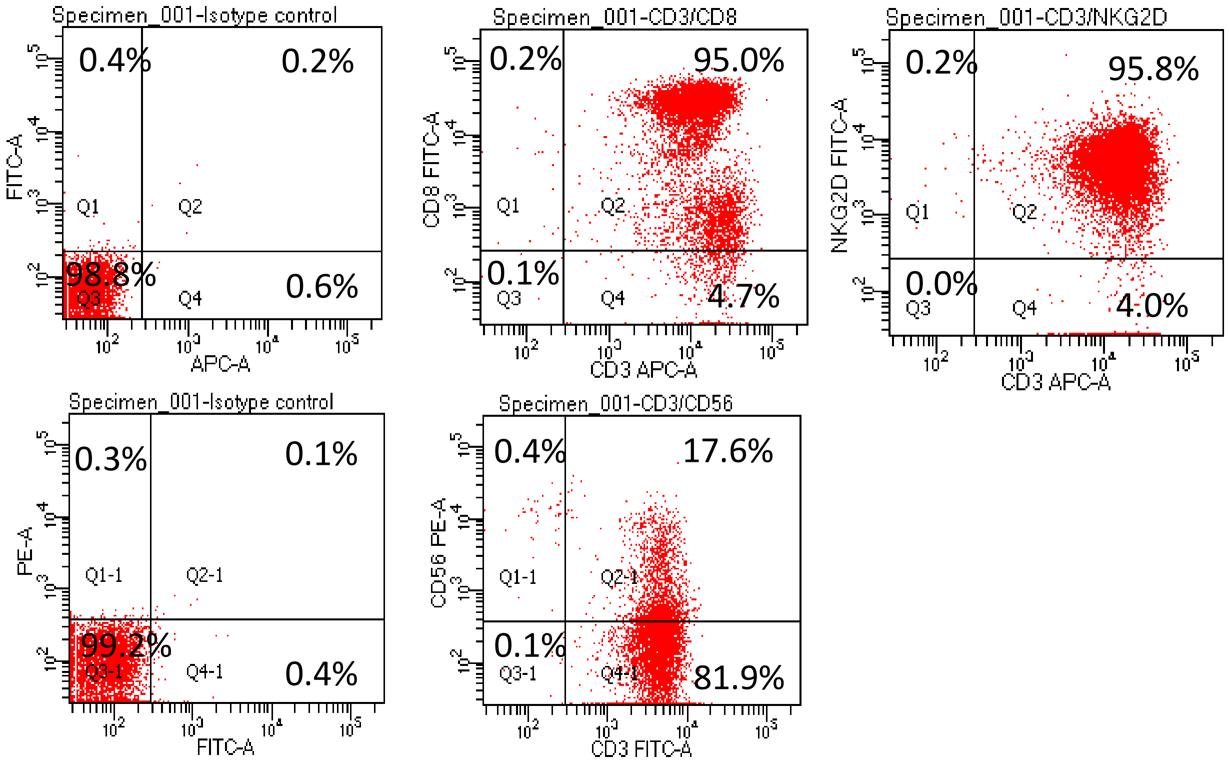

Case 27

DC markers

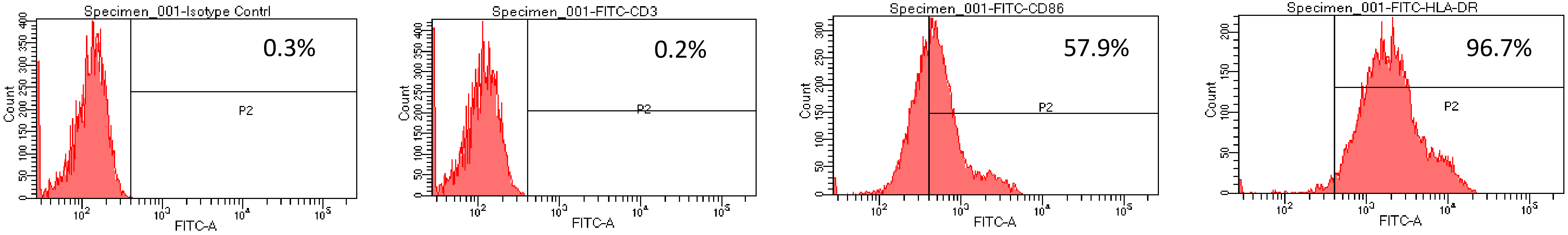

DC-CIK markers

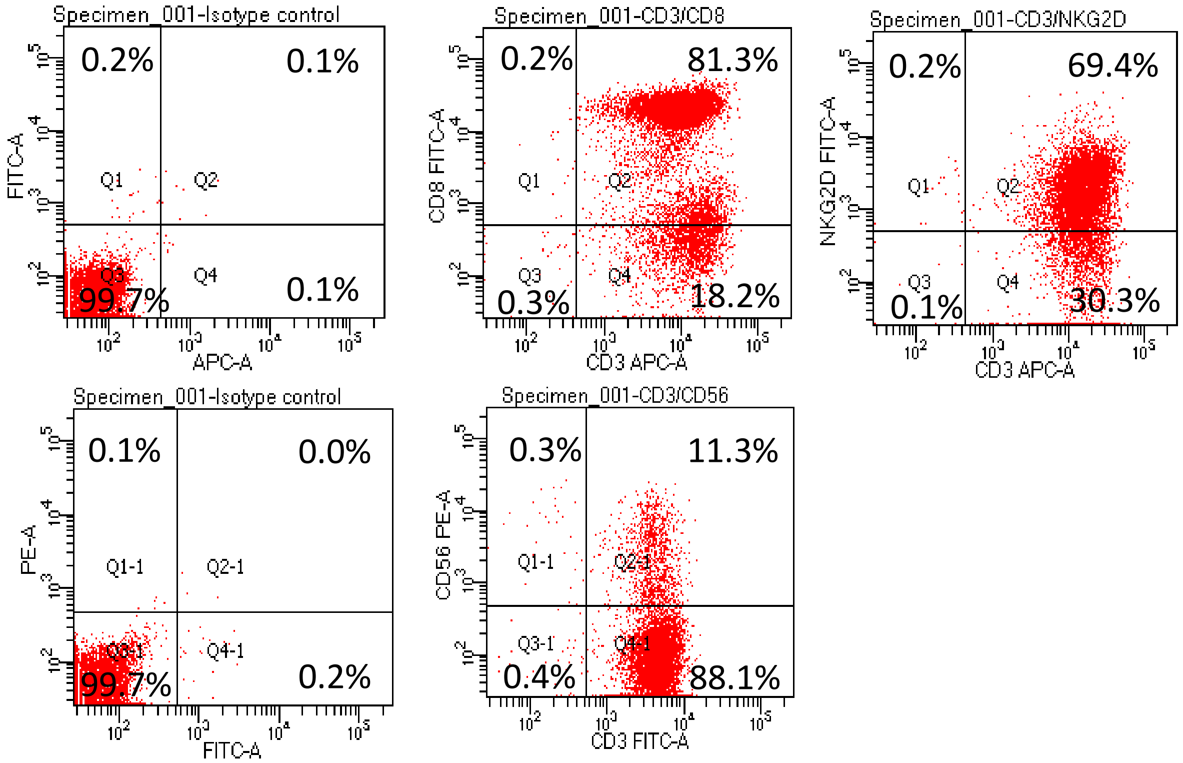

Supplement: Supplementary 1 — Patient’s allocation algorithm. [file DataSheet_1.pdf]
