# Supplementary material for: Effect of autologous dendritic cell cytokine-induced killer on refractory metastatic colorectal cancer: a matched case–control comparative study
Source: Front Immunol. 2024 Feb 27;15:1329615. doi: 10.3389/fimmu.2024.1329615 (PMC10927724; doi:10.3389/fimmu.2024.1329615)
Supplement: Supplementary file 2 [file DataSheet_2.pdf]

Supplement 2. Tumor response by (A) RESCIST (Response Evaluation Criteria in Solid Tumor), (B) CEA change from baseline to 8 weeks after therapy, compared between DC-CIK cases and controls

(A)

RECIST	**DC-CIK cases**	**Controls**
CR	0	0
PR	2	0
SD	17	15
PD	8	12
**Disease control rate**	**70.4%**	**55.6%**
P-value	0.232	

(B)

CEA change	**DC-CIK cases**	**Controls**
Decreased > 50%	6	2
Decreased < 50%	5	4
Increased	16	21
**CEA reduction rate**	**40.7%**	**22.2%**
P-value	0.248	
