# Supplementary figures and images for: Effect of autologous dendritic cell cytokine-induced killer on refractory metastatic colorectal cancer: a matched case–control comparative study
Source: Front Immunol. 2024 Feb 27;15:1329615. doi: 10.3389/fimmu.2024.1329615 (PMC10927724; doi:10.3389/fimmu.2024.1329615)

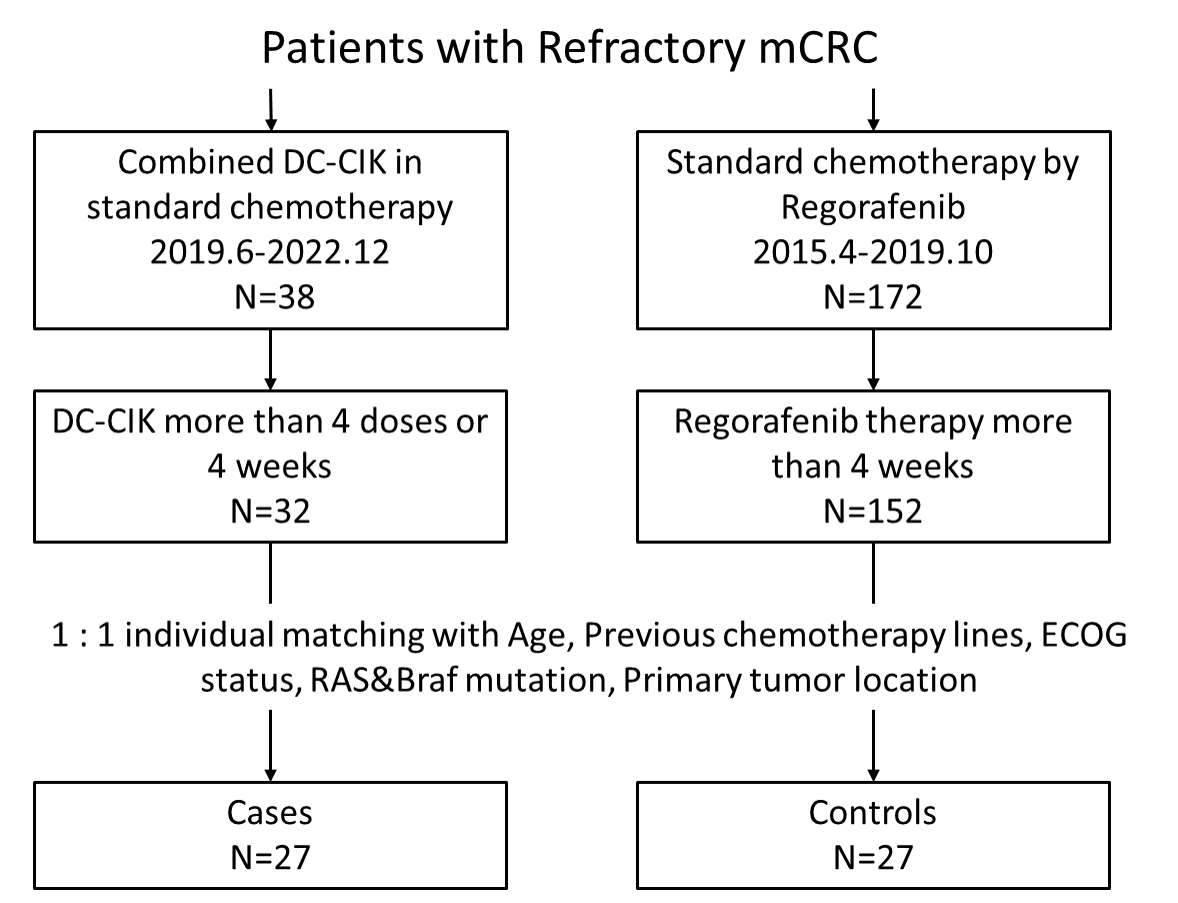

Supplement: Supplementary file 3 [file Image_1.tif]
